# Evaluation of an ^131^I-labeled HER2-specific single domain antibody fragment for the radiopharmaceutical therapy of HER2-expressing cancers

**DOI:** 10.1038/s41598-022-07006-9

**Published:** 2022-02-22

**Authors:** Yutian Feng, Rebecca Meshaw, Darryl McDougald, Zhengyuan Zhou, Xiao-Guang Zhao, Stephen A. Jannetti, Robert E. Reiman, Erica Pippen, Robin Marjoram, Jeffrey L. Schaal, Ganesan Vaidyanathan, Michael R. Zalutsky

**Affiliations:** 1 Department of Radiology, Duke University Medical Center, Durham, NC USA; 2 Cereius Inc, Durham, NC USA

**Keywords:** Nuclear chemistry, Targeted therapies, Breast cancer

## Abstract

Radiopharmaceutical therapy (RPT) is an attractive strategy for treatment of disseminated cancers including those overexpressing the HER2 receptor including breast, ovarian and gastroesophageal carcinomas. Single-domain antibody fragments (sdAbs) exemplified by the HER2-targeted VHH_1028 evaluated herein are attractive for RPT because they rapidly accumulate in tumor and clear faster from normal tissues than intact antibodies. In this study, VHH_1028 was labeled using the residualizing prosthetic agent *N*-succinimidyl 3-guanidinomethyl 5-[^131^I]iodobenzoate (*iso*-[^131^I]SGMIB) and its tissue distribution evaluated in the HER2-expressing SKOV-3 ovarian and BT474 breast carcinoma xenograft models. In head-to-head comparisons to [^131^I]SGMIB-2Rs15d, a HER2-targeted radiopharmaceutical currently under clinical investigation, *iso*-[^131^I]SGMIB-VHH_1028 exhibited significantly higher tumor uptake and significantly lower kidney accumulation. The results demonstrated 2.9 and 6.3 times more favorable tumor-to-kidney radiation dose ratios in the SKOV-3 and BT474 xenograft models, respectively. *Iso*-[^131^I]SGMIB-VHH_1028 was prepared using a solid-phase extraction method for purification of the prosthetic agent intermediate Boc_2_-*iso*-[^131^I]SGMIB that reproducibly scaled to therapeutic-level doses and obviated the need for its HPLC purification. Single-dose (SKOV-3) and multiple-dose (BT474) treatment regimens demonstrated that *iso*-[^131^I]SGMIB-VHH_1028 was well tolerated and provided significant tumor growth delay and survival prolongation. This study suggests that *iso*-[^131^I]SGMIB-VHH_1028 is a promising candidate for RPT of HER2-expressing cancers and further development is warranted.

## Introduction

Radiopharmaceutical therapy (RPT) has emerged as an attractive strategy for cancer treatment, particularly for patients with metastatic disease^1,2^. Because RPT is biologically targeted, it offers the exciting prospect of significantly increasing patient survival with fewer side effects than might be encountered with conventional external beam radiation^3^. RPT requires the judicious combination of a radioactive atom and tumor targeting vector, as well as an appropriate method for linking the two^4^. Regarding the targeting vehicle, rapid normal tissue clearance and tumor penetration are critical for ensuring the high therapeutic index necessary for successful RPT. Camelid-derived 13–15 kDa single domain antibody fragments (sdAb; also known as nanobodies or VHH molecules) provide an ideal platform as they exhibit these attractive properties^5^.

With sdAbs as well as engineered scaffold proteins of similar molecular weight, the challenge is to achieve high tumor uptake while avoiding excessive accumulation of radioactivity in the kidneys^6^. While the residualizing properties of radiometals help with the former, they can exacerbate the later, resulting in renal activity levels that would be unacceptable for RPT^7,8^. This limitation has commonly been associated with radiometals; however, it is not exclusive to these radio-conjugates. Similar difficulties were encountered with an sdAb radioiodinated using a highly residualizing prosthetic agent that contained multiple negatively charged D-amino acids^9^. Much more favorable results were obtained when sdAbs were labeled using *N*-succinimidyl 4-guanidinomethyl 3-[^131^I]iodobenzoate ([^131^I]SGMIB)—an agent combining the properties of good tumor residualization with radiolabeled catabolites that undergo rapid renal excretion^10,11^.

The human epidermal growth factor receptor type 2 (HER2) has been the most widely investigated molecule for targeting radiolabeled sdAbs to tumors^12^. This transmembrane receptor is frequently expressed on breast, gastric and ovarian carcinomas^13^. Moreover, HER2 overexpression is a harbinger of lower disease-free survival and metastatic disease^14^. Particularly for metastases to the brain, a common and frequently lethal manifestation in patients with HER2-positive breast cancer^15^, the smaller size of sdAbs compared with existing HER2-targeted intact antibodies and their drug conjugates could be advantageous^16,17^.

The potential advantages of sdAbs as a platform for developing HER2-targeted RPT agents have been summarized by Altunay et al.^12^ with a more general discussion of the rationale for developing radiolabeled sdAbs for RPT outlined in a recent review^5^. Although HER2-targeted whole antibodies (mAbs) provide high and prolonged uptake in tumor, their slow clearance from normal tissues compromises their potential for RPT as does their poor tumor penetration^12^. On the other hand, sdAbs can be developed with equivalent affinity as whole mAbs, exhibit rapid clearance from normal tissues, and in some cases, can achieve tumor uptake nearly as high as with intact mAbs^11^. The primary disadvantage of sdAbs for RPT is their high kidney uptake, which as noted above, has generally been more pronounced with radiometals than with radioiodine in preclinical models^5,12^.

The first clinical evaluation of a radiolabeled HER2-targeted sdAb ultimately intended for RPT recently was reported^18^. Six healthy volunteers and three HER2-positive breast cancer patients received ~ 38 MBq of VHH1 (also known as 2Rs15d) labeled using [^131^I]SGMIB and acceptable radiation dosimetry and toxicity profiles were observed. Selection of [^131^I]SGMIB-2Rs15d for clinical translation was based on preclinical studies demonstrating good tumor targeting and favorable biodistribution as well as therapeutic potential in HER2-expressing subcutaneous xenograft models^19^. Although better therapeutic responses were observed with its ^177^Lu-labeled counterpart, [^131^I]SGMIB-2Rs15d was selected for further development because it offered the possibility of achieving tumor control at a dosing regimen far less likely to cause renal toxicity^19,20^.

2Rs15d binds to HER2 Domain I, avoiding competition with trastuzumab (Domain IV) and pertuzumab (Domain II). However, consistent with this, only modest internalization and trapping of [^131^I]SGMIB-2Rs15d by HER2-expressing carcinoma cells was observed^19^. Although not as critical a factor as with HER2 targeted antibody–drug conjugates, combination of an sdAb that binds to a HER2 domain that triggers internalization with a residualizing labeling agent could be an attractive strategy for RPT. Previously, we evaluated the potential utility of anti-HER2 sdAb 5F7^9^, which binds to the same HER2 Domain IV epitope as ado-trastuzumab emtansine^21^, as a delivery vehicle for the development of HER2 RPT agents. As expected, a high degree of internalization in vitro and tumor localizing capacity in vivo were observed when 5F7 was labeled using [^131^I]SGMIB^11^. Surprisingly, labeling with structurally similar *N*-succinimidyl 3-guanidinomethyl 5-[^131^I]iodobenzoate (*iso*-[^131^I]SGMIB) resulted in significantly higher tumor uptake, more rapid renal clearance and less dehalogenation than observed with [^131^I]SGMIB-5F7^22^, making *iso*-[^131^I]SGMIB-5F7 of interest for RPT.

Because 5F7 has a lysine conjugation labeling site within one of its CDR regions^23^, compromised HER2 binding could occur, particularly when labeled at the higher specific activities that may be required for clinical RPT. For this reason, sdAb VHH_1028 was created and engineered to contain no lysine conjugation sites in its CDR regions. Herein, we have evaluated the characteristics of the novel *iso*-[^131^I]SGMIB-VHH_1028 conjugate and its potential utility for RPT of HER2-expressing cancers.

## Results

### VHH_1028

Characterization of the anti-HER2 VHH_1028 including its amino acid sequence, and analysis by SDS-PAGE and HPLC are presented in Supplementary Fig. S1. Size-exclusion HPLC–MS of VHH_1028 showed a single peak with a molecular weight of 12,844.0 Da within error of the theoretical value (12,844.2 Da). ELISA Analysis of VHH_1028 binding affinity, specificity and species cross reactivity is summarized in Supplementary Fig. S2.

### Synthesis and binding affinity of unlabeled *iso*-SGMIB-VHH_1028

Reaction of VHH_1028 with *iso*-SGMIB provided a mono-substituted conjugate, isolated in 25% yield by HIC HPLC, which showed a single peak with a molecular weight of 13,147.6 Da (calculated value 13,146.4 Da) on size-exclusion HPLC–MS. Sensorgrams and kinetic data from surface plasmon resonance (SPR) experiments demonstrated an affinity constant of 0.21 nM for binding of VHH_1028 and *iso*-SGMIB-VHH_1028 to HER2 (Supplementary Fig. S3).

### Scaled-up ***iso***-[^131^I]SGMIB-VHH_1028 production

**Figure 1:**
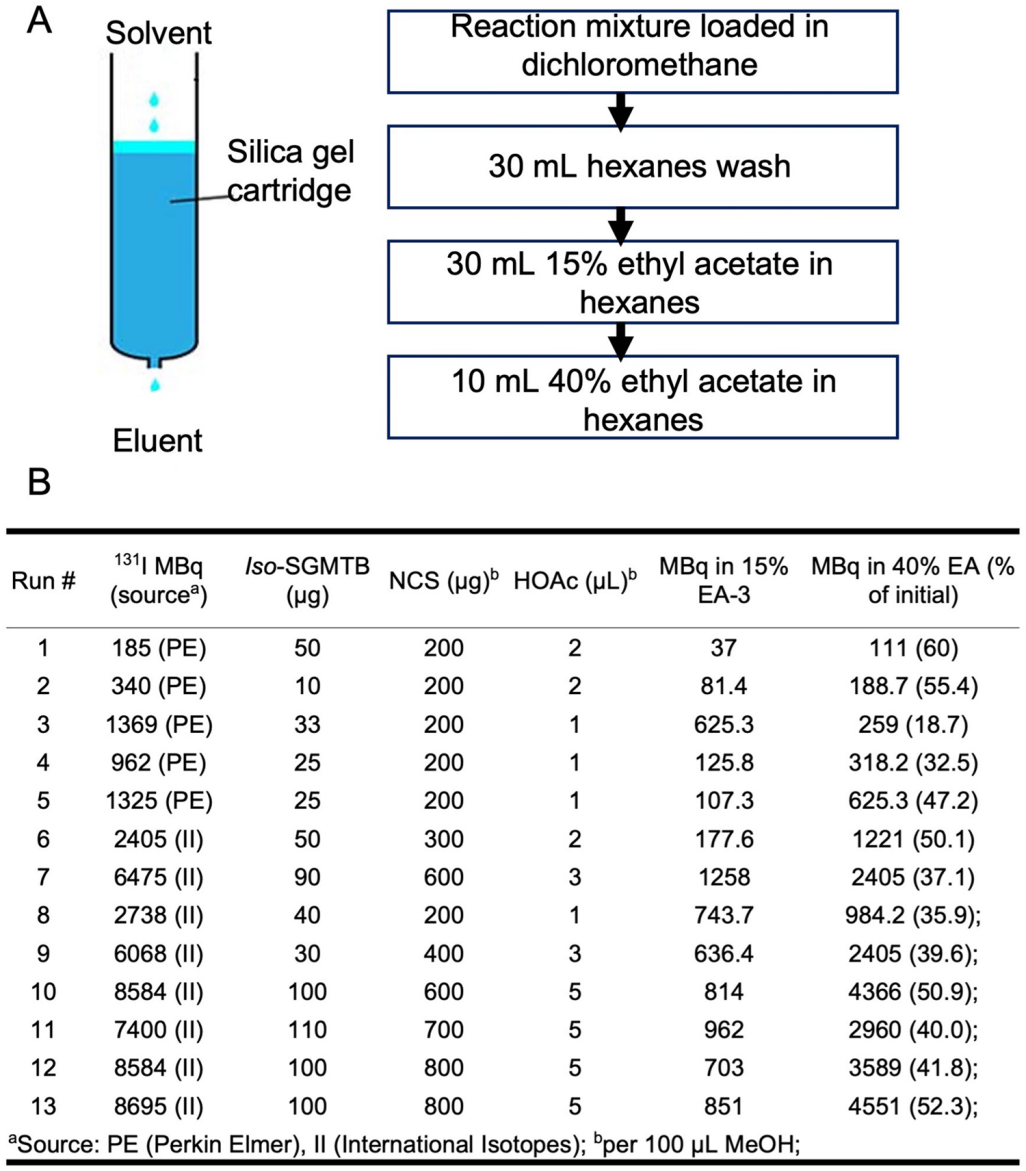
Scale-up production method for *iso*-[^131^I]SGMIB. (**A**) Description of the Silica Gel cartridge method for purification. (**B**) Radiolabeling and purification parameters.

Details for the streamlined synthesis and purification using the solid-phase extraction (SPE) method are shown in Fig. 1A and the experimental details for each synthesis are given in Fig. 1B. A total of 13 syntheses of Boc_2_-*iso*-[^131^I]SGMIB using the SPE method were performed with a radiochemical yield (RCY) of 48.5 ± 5.6%. With the SPE method, labeling was scaled successfully with consistent RCY from initial ^131^I activity levels from 0.19 GBq up to 8.7 GBq. The radiochemical purity (RCP) of Boc_2_-*iso*-[^131^I]SGMIB, determined by normal phase HPLC, was > 90% except in some cases when analysis was done > 4 h after the syntheses. VHH_1028 was labeled conjugated with *iso*-[^131^I]SGMIB in 39.8 ± 11.2% RCY; *iso*-[^131^I]SGMIB-VHH_1028 had a RCP of 96.7 ± 8.1% by SDS-PAGE/phosphor imaging. Immunoreactivity and cell-based affinity assays were not performed on all batches for practical reasons. The *iso*-[^131^I]SGMIB-VHH_1028 immunoreactive fraction was 81.9 ± 10.1% (n = 6) and its K_d_ values for binding to HER2-expressing SKOV-3 cells for three therapy-level batches were 1.5 ± 0.3 nM, 2.9 ± 0.5 nM and 2.6 ± 0.5 nM. A maximum of 5.4 GBq of *iso*-[^131^I]SGMIB and 1.2 GBq of *iso*-[^131^I]SGMIB-VHH_1028 were produced. The molar activity of *iso*-[^131^I]SGMIB-VHH_1028 ranged from 0.97 to 29.38 GBq/µmol. Batch 7 (Fig. 1B) was used for the single-dose therapy study and Batches 10–13 were used for the multiple-dose therapy study.

### In vitro stability

The in vitro stability of *iso*-[^131^I]SGMIB-VHH_1028 produced from Batch 8 was determined at 4 °C for up to 48 h. The radiochemical purity determined by SDS-PAGE was 97%, 96%, 90% and 89% at 18 h, 24 h, 42 h and 48 h, respectively (Supplementary Fig. S4).

### Cell uptake, retention and internalization

**Figure 2:**
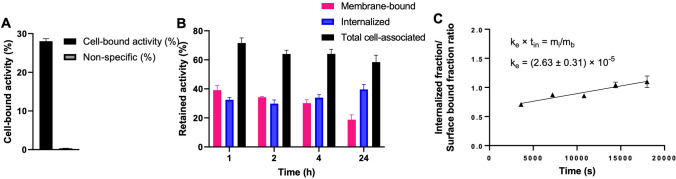
Cell uptake, retention and internalization assays for *iso*-[^131^I]SGMIB-VHH_1028 on HER2-positive BT474 breast carcinoma cells. (**A**) Total cell-bound activity and non-specific binding, measured by co-incubation with 100-fold excess of trastuzumab, after a 1 h incubation at 4 °C. (**B**) Retention of ^131^I activity after incubation at 37 °C for various times expressed as membrane-bound, internalized and total cell-associated activity; corrected for nonspecific binding by co-incubation with trastuzumab and (**C**) Internalization rate determined by plotting the ratio of internalized fraction over surface bound fraction, corrected for nonspecific binding, over time; internalization rate constant (k_e_) derived by linear regression (R^2^ = 0.8471). Surface-bound % activity and internalized % activity are presented as the percentages of initially cell-bound radioactivity after an initial incubation at 4 °C in these compartments. Surface-bound + internalized + supernatant = 100% at all time points.

Results for the uptake, retention and internalization of *iso*-[^131^I]SGMIB-VHH_1028 on BT474 cells are shown in Fig. 2. Binding of *iso*-[^131^I]SGMIB-VHH_1028 to BT474 cells after incubation at 4 °C for 1 h was 28.0 ± 0.7% of input activity, which was reduced to 0.33 ± 0.4% when cells were co-incubated with trastuzumab (Fig. 2A), confirming the HER2 specificity of cell binding. Between 64 and 71% that was bound after incubation at 4 °C for 1 h was retained by the cells with a gradual increase in intracellular activity observed during the 24-h experiment at 37 °C (Fig. 2B). The internalization rate constant, k_e_, for *iso*-[^131^I]SGMIB-VHH_1028 on BT474 cells was determined by linear regression of the ratio between internalized and membrane-bound activity, corrected for binding in the presence of an excess of trastuzumab (0.39–0.40%) , A k_e_ value of (2.63 ± 0.31) × 10^–5^ (s^-1^) (95% confidence interval: R^2^ = 0.8471) was calculated (Fig. 2C), which was similar to that measured previously for trastuzumab on this cell line^24^.

### Biodistribution studies

The biodistribution of *iso*-[^131^I]SGMIB-VHH_1028 and [^131^I]SGMIB-2Rs15d was determined in identical fashion in xenografted mice from the same cohort and randomized between the two radio-conjugates. The results obtained in NOD SCID mice bearing BT474 breast carcinoma xenografts are presented in Tables 1 and 2. Tumor uptake of *iso*-[^131^I]SGMIB-VHH_1028 was higher than that for [^131^I]SGMIB-2Rs15d at all time points with the difference being significant (*P* = 0.0056–0.0296) except at 1 h (27.6 ± 10.0% ID/g vs. 17.1 ± 6.0% ID/g; *P* = 0.0789). At 24 h, a more than two-fold tumor delivery advantage for *iso*-[^131^I]SGMIB-VHH_1028 was observed (14.0 ± 6.0% ID/g vs. 5.7 ± 1.3% ID/g). Except in the kidneys, clearance of both ^131^I-labeled sdAbs from normal tissues was rapid with minor differences in tissue retention observed in some cases. The activity levels in the kidneys for *iso*-[^131^I]SGMIB-VHH_1028 were 48.3 ± 6.9% ID/g at 1 h, 3.8 ± 0.9% ID/g at 4 h (*P* = 0.0067) and 1.3 ± 0.2% ID/g at 8 h. These values were significantly lower than those observed for [^131^I]SGMIB-2Rs15d, which were 74.1 ± 14.2% ID/g at 1 h (*P* = 0.0065), 15.4 ± 7.1% ID/g at 4 h (*P* = 0.0067) and 7.1 ± 4.9% ID/g at 8 h (*P* = 0.0295). The biodistribution results obtained in athymic mice with subcutaneous SKOV-3 tumors with the two ^131^I-labeled sdAbs are presented in Supplementary Tables S1 and S2. Although the magnitude of the %ID/g values were different between the two animal host-xenograft models, the differences were nearly identical, with *iso*-[^131^I]SGMIB-VHH_1028 exhibiting significantly higher tumor uptake and significantly lower kidney activity than that observed for [^131^I]SGMIB-2Rs15d.

**Table 1:** Biodistribution of [^131^I]SGMIB-2Rs15d in NOD SCID mice bearing BT474 xenografts.

Percent injected dose per gram of tissue^a^					
Tissues	1 h	4 h	8 h	16 h	24 h
Liver	5.3 ± 0.7	3.5 ± 0.8	2.2 ± 0.1	0.1 ± 0.0	0.0 ± 0.0
Spleen	1.2 ± 0.3	1.5 ± 0.9	0.6 ± 0.2	0.1 ± 0.0	0.0 ± 0.0
Lungs	2.8 ± 0.8	2.5 ± 1.0	1.2 ± 0.4	1.3 ± 0.7	0.1 ± 0.1
Heart	0.7 ± 0.3	0.3 ± 0.2	0.2 ± 0.1	0.0 ± 0.0	0.0 ± 0.0
Kidneys	74.1 ± 14.2	15.4 ± 7.1	7.1 ± 4.9	0.6 ± 0.3	0.5 ± 0.2
Stomach	1.1 ± 0.4	1.0 ± 0.8	0.9 ± 0.5	0.3 ± 0.3	0.1 ± 0.0
Thyroid	0.4 ± 0.3	0.4 ± 0.2	0.3 ± 0.2	0.1 ± 0.0	0.1 ± 0.1
Sm. Int	1.1 ± 0.3	1.4 ± 0.7	2.4 ± 4.0	0.3 ± 0.3	0.1 ± 0.0
Lg. Int	0.3 ± 0.1	2.2 ± 1.8	5.0 ± 3.6	0.9 ± 0.5	0.2 ± 0.1
Muscle	0.4 ± 0.1	0.1 ± 0.1	0.1 ± 0.0	0.0 ± 0.0	0.0 ± 0.0
Blood	1.5 ± 0.5	0.8 ± 0.2	0.5 ± 0.3	0.0 ± 0.0	0.0 ± 0.0
Bone	0.5 ± 0.2	0.2 ± 0.1	0.1 ± 0.0	0.0 ± 0.0	0.0 ± 0.0
Brain	0.1 ± 0.0	0.0 ± 0.0	0.0 ± 0.0	0.0 ± 0.0	0.0 ± 0.0
Tumor	17.1 ± 6.0	17.7 ± 4.7	11.0 ± 2.3	8.5 ± 2.2	5.7 ± 1.3

^a^Mean ± SD (n = 5).

**Table 2:** Biodistribution of *iso*-[^131^I]SGMIB-VHH_1028 in NOD SCID mice bearing BT474 xenografts.

Percent injected dose per gram of tissue^a^					
Tissues	1 h	4 h	8 h	16 h	24 h
Liver	1.9 ± 0.2	0.6 ± 0.1	0.2 ± 0.0	0.1 ± 0.0	0.1 ± 0.0
Spleen	0.9 ± 0.2	0.3 ± 0.0	0.2 ± 0.0	0.0 ± 0.0	0.1 ± 1.0
Lungs	2.7 ± 0.3	0.9 ± 0.3	0.4 ± 0.1	0.5 ± 0.4	0.2 ± 0.1
Heart	1.0 ± 0.2	0.2 ± 0.0	0.2 ± 0.0	0.1 ± 0.0	0.0 ± 0.0
Kidneys	48.3 ± 6.9	3.8 ± 0.9	1.3 ± 0.2	0.8 ± 0.8	0.2 ± 0.1
Stomach	1.3 ± 0.6	2.7 ± 0.4	1.4 ± 0.9	0.2 ± 0.0	0.2 ± 0.1
Thyroid	1.1 ± 0.5	1.0 ± 0.3	1.0 ± 0.1	0.3 ± 0.2	0.2 ± 0.1
Sm. Int	1.0 ± 0.3	2.0 ± 0.6	0.8 ± 0.3	0.6 ± 0.4	0.2 ± 0.1
Lg. Int	0.5 ± 0.2	4.4 ± 3.4	3.3 ± 1.3	3.0 ± 1.8	1.2 ± 0.8
Muscle	0.4 ± 0.1	0.2 ± 0.1	0.2 ± 0.1	0.1 ± 0.0	0.0 ± 0.0
Blood	2.6 ± 1.1	0.7 ± 0.1	0.8 ± 0.7	0.1 ± 0.0	0.0 ± 0.0
Bone	0.7 ± 0.2	0.4 ± 0.2	0.1 ± 0.1	0.1 ± 0.0	0.1 ± 0.0
Brain	0.1 ± 0.0	0.1 ± 0.0	0.0 ± 0.0	0.0 ± 0.0	0.0 ± 0.0
Tumor	27.6 ± 10.0	26.8 ± 6.1	16.7 ± 3.0	32.6 ± 18.4	14.0 ± 6.0

^a^Mean ± SD (n = 5).

### Radiation dosimetry

We next used these biodistribution data to estimate the radiation doses that would be received by the tumor and the kidney in a therapeutic efficacy study performed in mice at a hypothetical dose of 37 MBq. The goodness of fit (R^2^ value) for tumor uptake of *iso*-[^131^I]SGMIB-VHH_1028 was 0.9921 for the BT474 model and 0.8764 for SKOV-3 model; those for [^131^I]SGMIB-2Rs15d were 0.9675 (BT474) and 0.8254 (SKOV-3). Nonlinear regression fit for kidney uptake was performed with R^2^ values of 0.9779 (BT474) and 0.9032 (SKOV-3) for *iso*-[^131^I]SGMIB-VHH_1028 and 0.9427 (BT474) and 0.9179 (SKOV-3) for [^131^I]SGMIB-2Rs15d. In the SKOV-3 model, at an equivalent radiation dose to the kidney (presumed dose limiting organ), 2.9 times more radiation dose could be delivered to tumor with *iso*-[^131^I]SGMIB-VHH_1028 compared to [^131^I]SGMIB-2Rs15d (Table 3). Likewise, 6.3 times more radiation dose could be delivered to BT-474 tumors with *iso*-[^131^I]SGMIB-VHH_1028 compared to [^131^I]SGMIB-2Rs15d. Using the BT474 model data sets, radiation absorbed doses and effective doses were estimated for humans using the MIRD method (Supplementary Table S3). The effective doses were 0.148 mSv/MBq and 0.105 mSv/MBq for *iso*-[^131^I]SGMIB-VHH_1028 and [^131^I]SGMIB-2Rs15d, respectively, and the estimated radiation absorbed doses to the kidneys were 0.25 and 0.44 mSv/MBq, respectively.

**Table 3:** Absorbed cumulative radiation dose estimates of [^131^I]SGMIB-2Rs15d and *iso-*[^131^I]SGMIB-VHH_1028 to kidneys and tumors for a hypothetical dose of 37 MBq.

Animal model	sdAb	Prosthetic agent	Cumulative activity (µCi*h/g)		Radiation dose (cGy)	
			Kidney	Tumor	Kidney	Tumor
SKOV-3	VHH_1028	*iso*-[^131^I]SGMIB	445	2326	192	1003
	2Rs15d	[^131^I]SGMIB	849	1541	366	665
BT474	VHH_1028	*iso*-[^131^I]SGMIB	1000	8805	431	3798
	2Rs15d	[^131^I]SGMIB	2127	2965	917	1279

Estimates were based on biodistribution data in Tables 1 and 2. Cumulative radioactivity were calculated using the trapezoid integration method by calculating area under the activity/g tissue curves, extrapolating to when negligible activity remained. They were converted to absorbed cumulative dose by multiplying the equilibrium absorbed dose constant for ^131^I, 0.4313 g*cGy/(µCi*h).

### Therapeutic efficacy of ***iso***-[^131^I]SGMIB-VHH_1028

**Figure 3:**
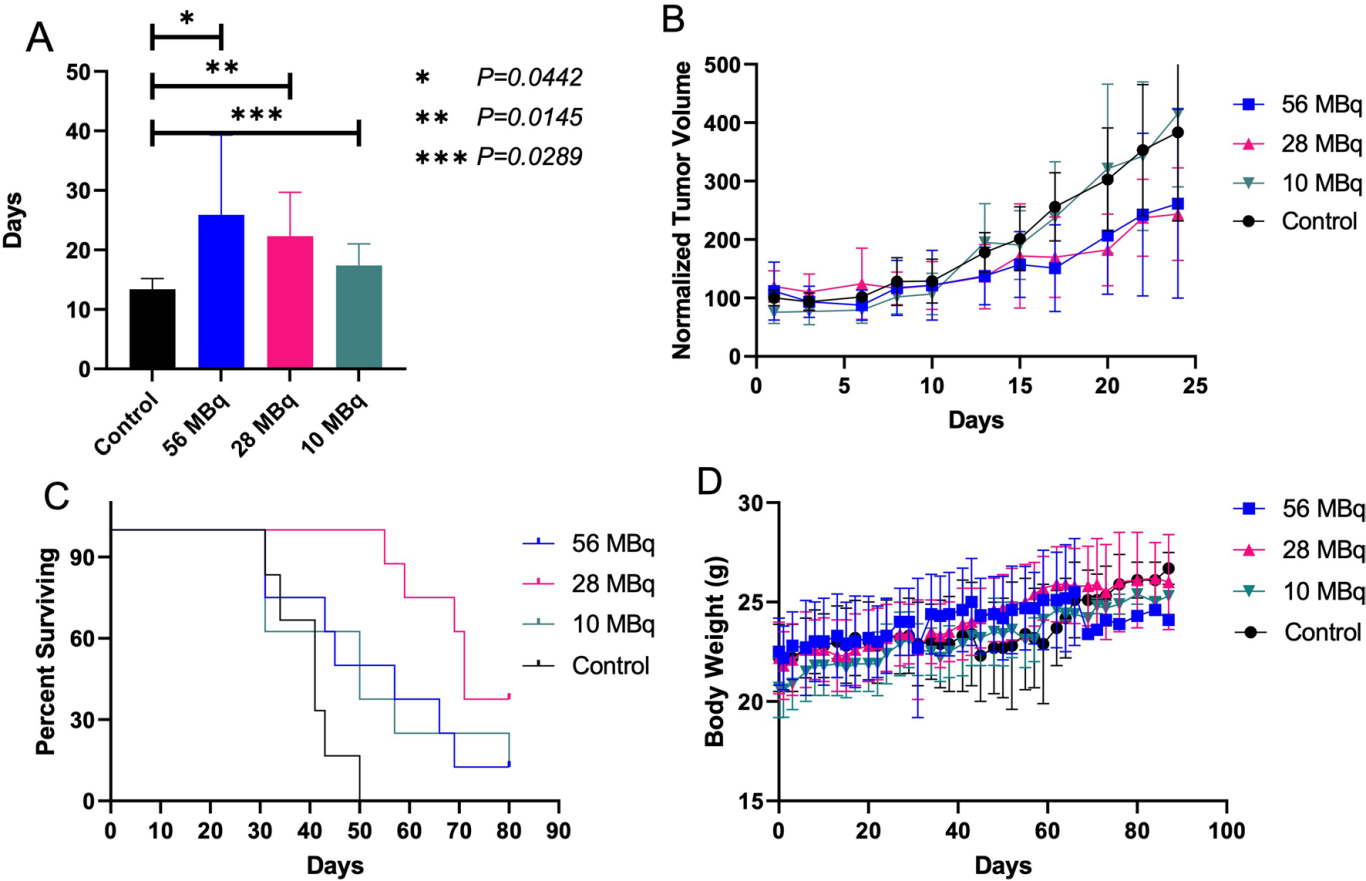
Therapeutic efficacy of single dose *iso*-[^131^I]SGMIB-VHH_1028 in mice with SKOV-3 xenografts. (**A**) Days to reach 200% of initial tumor volume for each group. Student *t-test* performed between treatment groups and control group. (**B**) Normalized tumor volumes in each group during first 25 days post treatment. (**C**) Kaplan–Meier survival plot. (**D**) Body weight in each group for the duration of the study.

Tumor volumes at treatment and dosing details for the single-dose therapy study in athymic mice with SKOV-3 xenografts are summarized in Supplementary Table S4. As shown in Fig. 3A, *iso*-[^131^I]SGMIB-VHH_1028 treatment resulted in significant tumor growth delay with just a single dose. Days to reach 200% of initial tumor volume for treatment groups were significantly longer than the control group at 56 MBq (*P* = 0.0442), 28 MBq (*P* = 0.0145), and 10 MBq doses (*P* = 0.0289). Tumor volumes (mm^3^) for individual mice are presented in Supplementary Fig. S5. When tumor volumes in each animal were normalized to the volume at the time of treatment (Fig. 3B) and analyzed by paired *t* tests, significant tumor growth delay was observed as early as Day 17 with *P* values of 0.0199, 0.0081, and 0.0357 for high, medium, and low dose, respectively, *vs.* control. Differences in tumor growth delay among treatment groups were not statistically significant. Median survival was 51, 71, 50 and 41 d (Fig. 3C) for the 56, 28 and 10 MBq doses, and control group, respectively. Significant survival benefit compared with controls was observed only for the medium dose group according to both the Log-rank (Mantel-Cox) test (*P* < 0.0001) and Gehan-Breslow-Wilcoxon test (*P* = 0.0002). No significant differences in body weight were observed between treatment and control groups (Fig. 3D), and no signs of overt toxicity were observed.

**Figure 4:**
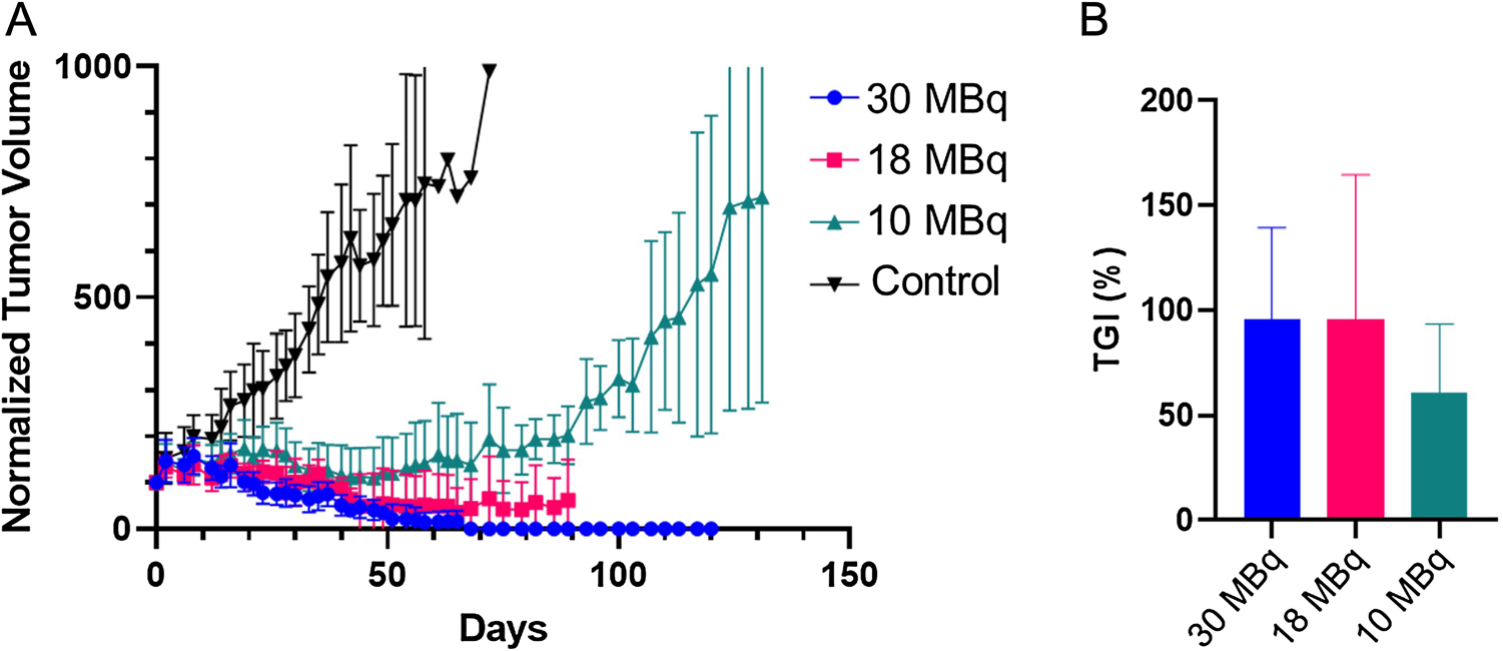
Effect of four doses of *iso*-[^131^I]SGMIB-VHH_1028 on tumor growth of BT474 xenografts in SCID mice. (**A**) Normalized tumor volume as a function of time. (**B**) Tumor growth inhibition (TGI%) of the treatment groups.

Next, NOD SCID mice with BT474 xenografts were treated in a multiple-dose therapy experiment. Tumor volumes at treatment and dosing details are summarized in Supplementary Table S5. Significant and dose-dependent tumor growth inhibition was observed across all *iso*-[^131^I]SGMIB-VHH_1028 groups (Fig. 4A) with a tumor growth inhibition (TGI%) of 95.7 ± 43.7%, 95.4 ± 69.2%, and 61.1 ± 32.2% for the 30, 18, and 10 MBq dose groups, respectively (Fig. 4B). Paired *t* tests indicated significant tumor growth inhibition for all treatment groups compared with the control group (*P* < 0.0001) as well as between dose levels (*P* < 0.0001 for 30 vs. 18 MBq, 30 vs. 10 MBq, and 18 vs. 10 MBq groups). Tumor volumes (mm^3^) for individual mice are presented in Supplementary Fig. S6.

**Figure 5:**
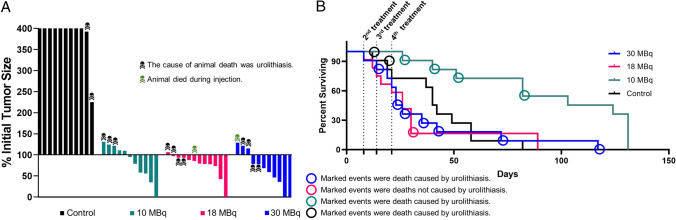
Evaluation of the therapeutic efficacy of four doses of *iso*-[^131^I]SGMIB-VHH_1028 in SCID mice with subcutaneous BT474 xenografts. (**A**) Maximum tumor response waterfall plot. (**B**) Kaplan–Meier survival plot. The 4 treatment times are as marked with the first given at Day 0. Animal deaths due to urolithiasis related to ER pellet implantation are indicated.

In the BT474 xenograft experiment, four mice exhibited complete responses (CR, 100% regression) during the study (Fig. 5A). In the 30 MBq group, 2 mice exhibited a CR and 6 had partial responses (PR, > 30% regression) for an 80% overall response rate (ORR). One CR also was observed in both the 18 and 10 MBq groups. Unfortunately, most mice developed complications from estrogen pellet-induced urolithiasis, a known complication of this model^25^, and died. Two mice in the control group, and 4, 9 and 7 in the 10, 18, and 30 MBq groups, respectively, died due to toxicity caused by urolithiasis as determined by necropsy. Supplementary Fig. S7 shows the anatomy of a normal mouse and the grossly enlarged, urolithiasis-blocked bladder of an untreated mouse from this study.

Median survival was 23, 26, 103 and 58 d for the 30, 18 and 10 MBq dose, and control groups, respectively (Fig. 5B). Significant survival benefit was observed only for the low dose group [*P* = 0.0007, log-rank (Mantel-Cox) test; *P* = 0.0041, log-rank test for trend; *P* = 0.0022, Gehan-Breslow-Wilcoxon]. It is important to note that the leading cause of death in these animals was due to estrogen pellet-induced urolithiasis complications (Fig. 5B). The normalized average body weights of treated mice were not significantly different from those of control mice (two-tailed paired *t*-test; 30 MBq vs controls (*P* = 0.794); 18 MBq vs. controls (*P* = 0.808); 10 MBq vs. controls (*P* = 0.237). No overt signs of acute or long-term treatment-related toxicity were observed.

## Discussion

RPT is gaining momentum as a strategy for cancer treatment^1,2^ and sdAbs have attractive characteristics for developing RPT agents including robust tumor accumulation combined with rapid normal tissue clearance^5^. For internalizing tumor-associated targets like HER2, the use of residualizing prosthetic agents for sdAb radioiodination has shown great potential and in some respects—notably, lower trapping of radioactivity in the kidneys—can offer substantive advantages compared with their radiometal-labeled counterparts^19,20^. A recent clinical study reported that ^131^I-GMIB-anti-HER2-VHH1 (*i.e.* [^131^I]SGMIB-2Rs15d) was well tolerated and selectively accumulated HER2 positive lesions^18^, and evaluation of its therapeutic efficacy is planned (NCT04467515). Thus, [^131^I]SGMIB-2Rs15d serves as a valuable benchmark for evaluating the potential utility of other combinations of prosthetic agents and anti-HER2 sdAbs as RPT agents.

Regarding current and future efforts directed at optimizing HER2-targeted sdAbs for RPT, at least with existing radiochemistry methods, there is a clear advantage for radiohalogens compared with radiometals for labeling sdAbs^8,20^. Thus, for a given molecular target, the most critical components are the properties of the prosthetic agent and those of the sdAb, and the interplay between the two. For radiohalogenation of proteins, two important design considerations that are largely independent of molecular target and binding epitope are minimizing dehalogenation in vivo and ensuring that radiolabeled catabolites that may be generated are rapidly excreted. Along with the goal of achieving intracellular trapping of radioiodine when used with internalizing proteins, these considerations guided the design of the original SGMIB reagent^10^. The importance of the residualizing capability of SGMIB and similar reagents likely depends on the degree to which internalization occurs. With HER2, reagents that bind to Domain 1 such as 2Rs15d and Domain II like pertuzumab internalize much less than those that bind to Domain IV exemplified by trastuzumab^19^. With modestly internalizing sdAbs, the need for a residualizing prosthetic agent may be lower while the prospect of achieving prolonged intracellular trapping of activity in tumor also will be lower, which could compromise therapeutic effectiveness. On the other hand, we speculate that sdAbs that target HER2 Domain IV and rapidly internalize are more challenging from a radiochemistry perspective because effective radionuclide residualization strategies become more important. However, an internalizing radioligand combined with intracellular trapping of the radionuclide provides an opportunity to achieve sustained irradiation of targeted cancer cells. Moreover, this minimizes loss of radiation dose due to dissociation from receptor on the cell membrane and also moves the site of radioactive decay closer to the cell nucleus, where it can be more effective.

Recently, we reported that the 1,3,5 isomer of SGMIB (*iso*-SGMIB) was a better residualizing prosthetic agent for labeling sdAbs, resulting in higher tumor uptake, less deiodination in vivo and lower activity levels in normal tissues including kidneys^22^. Likewise, an sdAb that targeted Domain IV of HER2, 5F7, exhibited excellent tumor targeting and internalizing properties^9,11,22,26–28^ that appeared advantageous compared to 2Rs15d^28,29^, although published head-to-head comparisons are lacking. However, the CDR loops of the 5F7 sdAb include a lysine, a potentially disadvantageous feature because conjugation labeling at this site could occur. At the higher activity levels required for RPT, this becomes more likely, potentially adversely affecting HER2 binding and subsequent internalization. To avoid this potential problem, VHH_1028 was designed and engineered to target HER2 Domain IV with nanomolar affinity but without potential conjugation sites present in its CDR loops. SPR assays confirmed that VHH_1028 and its 1:1 unlabeled *iso*-SGMIB-VHH_1028 conjugate gave identical K_d_ of 0.21 nM for HER2 binding.

Herein we have directly compared the biodistribution of [^131^I]SGMIB-2Rs15d and *iso*-[^131^I]SGMIB-VHH_1028 in two HER2-expressing xenograft models. A paired-label format (^131^I and ^125^I) was not used because of concerns that binding of 2Rs15d to Domain I might sterically interfere with access of VHH_1028 to Domain IV, which is closer to the cell surface; a preliminary experiment suggested that this could be an issue. Head-to-head comparisons were performed in single-label format with mice randomized between sdAbs and with the same time points evaluated for both on the same day.

With small proteins like sdAbs or peptides, the kidneys are the most likely dose limiting tissue^1,2,6–8^. The kidney uptake values and radiation absorbed doses for [^131^I]SGMIB-2Rs15d were similar to previously reported levels^19^ and lower than seen with 2Rs15d-radiometal conjugates^20,30–33^. In both xenograft models, *iso*-[^131^I]SGMIB-VHH_1028 exhibited significantly lower peak kidney levels and faster renal clearance than [^131^I]SGMIB-2Rs15d. Radiation dose calculations based on these data sets (Table 3) indicated that it should be possible to administer 2.1 and 1.9 times more radioactivity with *iso*-[^131^I]SGMIB-VHH_1028 than [^131^I]SGMIB-2Rs15d in the SCID/BT474 and athymic/SKOV-3 murine models, respectively, while maintaining the same radiation dose to the kidneys.

Tumor uptake and radiation absorbed doses for [^131^I]SGMIB-2Rs15d in both the SKOV-3 and BT474 xenograft models determined herein were comparable to those reported earlier; for example, we estimated a dose of 12.79 Gy to BT474 xenografts per 37 MBq, in good agreement with 11.88 Gy reported by D’Huyvetter et al*.* for BT474/M1 xenografts^19^. Tumor uptake of ^131^I activity was higher and more prolonged for *iso*-[^131^I]SGMIB-VHH_1028 than that for [^131^I]SGMIB-2Rs15d particularly in the BT474 xenograft model. Radiation doses per 37 MBq were also higher, 10.03 and 37.98 Gy, in the SKOV-3 and BT474 models, respectively. We speculate that this tumor delivery advantage might reflect the faster on-rate and more extensive internalization of VHH_1028 compared with 2Rs15d^19,31^, properties that should facilitate tumor binding and retention before the rapidly clearing VHH molecules can exit the blood pool. Taken together, the kidney and tumor radiation dose results suggest that *iso*-[^131^I]SGMIB-VHH_1028 could offer a nearly threefold higher therapeutic index in the SKOV-3 model and a 6.3-fold advantage in the BT474 model when compared with [^131^I]SGMIB-2Rs15d. Consequently, *iso*-[^131^I]SGMIB-VHH_1028 was selected for further investigation as a therapeutic radiopharmaceutical in both HER2-expressing animal models.

Previously, synthesis of *iso*-[^131^I]SGMIB was performed using a trimethyl tin precursor and involved a normal phase HPLC purification^21,26^. To avoid cumbersome HPLC, especially for working with high activity levels, a SPE method was developed by adapting a previously reported method used for the synthesis of a different prosthetic agent^34^. To facilitate separation of *iso*-[^131^I]SGMIB and the tin precursor, a tributyl tin analogue was used for the synthesis. The radiolabeling and SPE purification conditions evolved with each batch, in part because the amount of tin precursor and other reagents needed to be adjusted based on the ^131^I activity level and molar activity, and solvent composition. After loading the reaction mixture, the silica cartridge was washed with hexanes to remove any unreacted tin precursor. The activity from the 40% ethyl acetate in hexanes fraction alone, or in some cases, combined with the last 15% ethyl acetate/hexanes fraction, was used without further purification for VHH conjugation. Any radiochemical impurities, if present, should not adversely affect the conjugation reaction. A maximum of 5.4 GBq Boc_2_-*iso*-[^131^I]SGMIB has been produced by this method to date and deprotected nearly quantitatively with TFA to provide what is perhaps the highest amount of any ^131^I-labeled prosthetic agent produced to date.

Conjugation of *iso*-[^131^I]SGMIB to VHH_1028 followed a reported method^10,22,26^ and utilized a molar ratio of 1.2:1 VHH_1028:*iso*-[^131^I]SGMIB. Conjugation was performed at VHH_1028 concentrations of between 4 and 7 mg/mL. We sought to balance two factors: labeling proteins by active ester conjugation at higher protein concentration gives higher conjugation yields^35^; but a higher concentration also necessitates the use of lower total volumes, which could potentially decrease yield due to limited physical contact even when using conical bottom vials. It was found that 7 mg/mL and 30 µL volume provided the best results. The molar activity of the *iso*-[^131^I]SGMIB-VHH_1028 varied largely depending on the conjugation yields. Excellent radiochemical purity, immunoreactivity and binding affinity were obtained for the *iso*-[^131^I]SGMIB-VHH_1028 using *iso*-[^131^I]SGMIB purified via the SPE method. A maximum of 1.2 GBq was produced, which to the best of our knowledge is the highest amount reported for an ^131^I-labeled sdAb.

Initially, the therapeutic efficacy of *iso*-[^131^I]SGMIB-VHH_1028 was investigated in single dose format in athymic mice with SKOV-3 xenografts. A maximum of 56 MBq was administered and well tolerated; based on dosimetry calculations, 56 MBq only delivered an absorbed dose of 2.91 Gy to kidneys, well below the renal toxicity threshold of 23 Gy predicted from external beam therapy. Compared with controls, significant tumor growth delay was observed for the three treatment groups 15 days post treatment, with the highest treatment dose delivering an estimated 15.18 Gy dose to tumors. The study was terminated on day 87, with only the medium dose providing a statistically significant improvement in median survival with 3 of animals in that group being long-term survivors. No overt signs of toxicity were observed in any treatment groups, and no significant difference was observed in the average body weight among groups. Thus, the reason for the lack of significant survival benefit in the high-dose group is unclear.

The therapeutic efficacy of *iso*-[^131^I]SGMIB-VHH_1028 was further evaluated in SCID mice with BT474 subcutaneous xenografts in a multiple-dose treatment regimen. Based on the biodistribution data derived dosimetry calculations, each weekly treatment resulted in estimated radiation doses of 10.26, 18.47 and 30.79 Gy, respectively, for the 10 MBq, 18 MBq and 30 MBq dose groups. Encouragingly, 55%, 92% and 73% of animals in the 10 MBq, 18 MBq and 30 MBq dose groups, respectively, showed tumor regression after treatment with *iso*-[^131^I]SGMIB-VHH_1028. Tumor was not detectable in 4 mice in the treatment groups. More than 50% tumor regression was observed in 36% of mice in the high dose and 2 mice each from the low and medium dose groups. Comparison to the results reported for a [^131^I]SGMIB-2Rs15d^19^ is warranted but difficult as tumor growth inhibition results were not reported. In addition, our study utilized four treatments compared with their five-dose protocol. Finally, considerable differences in tumor size at treatment, a critical factor for RPT, exist between the two studies with the [^131^I]SGMIB-2Rs15d study utilizing a three times smaller initial tumor volume of 50 mm^3^^19^.

The primary cause of death in the BT474 model was urolithiasis, likely caused by the sustained-release estrogen pellet necessary for establishing these BT474 xenografts^25^. Sustained-release estrogen pellets can lead to widely varying serum estrogen levels^36,37^, which can decrease urinary frequency and as a result, cause urinary retention in mice^38,39^. In the current study, urolithiasis was confirmed at necropsy including in mice that did not receive the ^131^I-labeled sdAb (Supplementary Fig. S7). The median survival for the low dose group was 103 days, which had the fewest urolithiasis related deaths in the treatment groups. This was 45 days longer than that of the control group. An estimated radiation absorbed dose of 116, 209, and 349 cGy was delivered to kidneys from each treatment at the 10, 18 and 30 MBq doses, respectively. Renal toxicity in mice would not be expected at these dose levels, as the threshold for renal toxicity in mice is reported to be 24 Gy^40^; moreover, renal toxicity was not observed at similar kidney radiation doses with other RPT agents^19,41^. Urolithiasis was not observed at necropsy in the BT474 biodistribution study consistent with this experiment being performed much earlier after estrogen pellet implantation than the therapy study. However, this suggests that the kidney absorbed dose in therapy animals is likely higher than those calculated based on the biodistribution data due to estrogen pellet related, prolonged kidney clearance. That there was a trend for more urolithiasis related deaths in the treatment groups might indicate that a secondary effect of increased renal radiotoxicity derived from compromised excretion also played a role. Nevertheless, no signs of renal toxicity were observed in the surviving mice after 100 days. In future studies using the BT474 estrogen-dependent animal model, we plan to utilize other estrogen administration methods reported to avoid urolithiasis^36,42–44^.

Estimating radiation dosimetry for RPT agents in humans based on murine biodistribution data must be done with caution because of potential species-dependent differences in the interaction of the radiopharmaceutical and its molecular target. For example, differences in organ expression patterns between species occur with prostate specific membrane antigen (PSMA)^45^. Although these differences exist, radiation dosimetry calculations based on murine biodistribution data have been used to permit clinical translation of more than 10 different PSMA-targeted small molecules and antibodies. Regarding species-dependent differences in receptor recognition, drugs that target the human HER2 extracellular domain do not bind to murine HER2^46^ as was the case in the current study. Nonetheless, the radiation dosimetry estimates we calculated for the human for [^131^I]SGMIB-2Rs15d (Supplementary Table S3) are in reasonable agreement with those reported by D’Huyvetter et al.^18^ for this radioconjugate based on patient images. Notably, in the heart and lungs—two organs with relatively high HER2 expression in the human^47^—the radiation dose calculated from our mouse data was only 46% and 20% lower than that measured in humans^18^. On the other hand, in the kidneys, which have relatively low levels of HER2 expression in humans, the dose estimated from mouse biodistribution data was about 4 times lower than that measured in humans, which likely reflects species-dependent differences in renal clearance rates. We speculate that the targetless component of the distribution of these small proteins is the dominant feature in determining their tissue residence times rather than receptor binding consistent with the low absolute levels if HER2 expression in normal human tissues. As done by D’Huyvetter et al.^18^, prior to evaluating therapeutic efficacy, we plan to perform a Phase 0 imaging trial to determine the dosimetry of *iso*-[^131^I]SGMIB-VHH_1028 in humans.

## Conclusion

Encouraged by the advancement of the HER2-targeted [^131^I]SGMIB-2Rs15d to clinical trial^18^, we have developed *iso*-[^131^I]SGMIB-VHH_1028, which has incorporated several distinguishing design features: a prosthetic agent imparting more favorable in vivo characteristics and an anti-HER2 sdAb with higher HER2 binding affinity and better internalization. Head-to-head biodistribution experiments in two animal models confirmed that *iso*-[^131^I]SGMIB-VHH_1028 exhibited higher and more prolonged tumor accumulation, and lower kidney levels than [^131^I]SGMIB-2Rs15d. As a result, tumor-to-kidney radiation dose ratios for *iso*-[^131^I]SGMIB-VHH_1028 were 2.9 times higher in the SKOV-3 model and 6.3 times higher in the BT474 model. Therapeutic efficacy studies with *iso*-[^131^I]SGMIB-VHH_1028 showed good tumor growth inhibition and survival benefit. Moreover, single doses as high as 56 MBq were well tolerated, and radiation dosimetry calculations suggest that even higher activity levels could be administered without causing renal toxicity. With regard to the radiochemistry aspects of this study, Boc_2_-*iso*-[^131^I]SGMIB was produced at high activity levels utilizing a cartridge-based purification method obviating the need for HPLC. To the best of our knowledge, *iso*-[^131^I]SGMIB and *iso*-[^131^I]SGMIB-VHH_1028 were produced at higher activity levels than those previously reported for any ^131^I-labeled prosthetic agent and its protein conjugate. These radiochemistry procedures should facilitate clinical translation of *iso*-[^131^I]SGMIB-VHH_1028 even at the doses required for patient RPT. These methods should be applicable to labeling sdAbs and other small proteins with *iso*-[^131^I]SGMIB; however, preliminary experiments indicate that extension to the [^131^I]SGMIB isomer will be difficult due to sterically imposed constraints.

## Methods

### General

Details about the sources for materials and general procedures used in these experiments are presented in Supplementary Methods.

### VHH_1028

This anti-HER2 sdAb used in these studies was developed and provided by Cereius, Inc. Details about its production and purification, and characterization of affinity and specificity by ELISA are presented in Supplementary Materials.

### Synthesis and binding affinity of unlabeled *iso*-SGMIB-VHH_1028

*Iso*-SGMIB was synthesized as described^26^. VHH_1028 (0.09 µmol in 300 µL of sodium borate buffer, pH 8.5) was reacted with *iso*-SGMIB (0.27 µmol, in 5 µL DMSO) at 30 °C for 2 h. The 1:1 *iso*-SGMIB-VHH_1028 conjugate was isolated by HIC-HPLC (Supplementary Methods). *Iso*-SGMIB-VHH_1028 and VHH_1028 molecular weights were determined by SE-LC–MS mass spectrometry and their binding affinity (K_d_) to HER2-Fc protein was measured by SPR using a Biacore 3000 System.

### Scaled-up ***iso***-[^131^I]SGMIB-VHH_1028 production

To streamline *iso*-[^131^I]SGMIB-VHH_1028 production at high ^131^I activity levels, a solid-phase extraction (SPE) method was used in lieu of NP HPLC purification. The experimental procedure varied over the different syntheses; the optimized protocol is as follows:

A solution of Boc_2_-*iso*-SGMTB (^26^; 1.1–1.5 molar equivalents of ^131^I) in 100 µL of anhydrous methanol containing acetic acid (1–5%) and *N*-chlorosuccinimide (NCS) (2–8%) was added to the dried ^131^I activity (0.19–8.7 GBq). The vial was vortexed for 30 s, reacted at room temperature for 30 min, and then the methanol was evaporated using a nitrogen stream. To insure complete removal of methanol, co-evaporation with ethyl acetate (100 µL) was performed twice. The residual activity was reconstituted in dichloromethane (2 × 100 µL) and loaded onto an activated silica cartridge (650 mg; Waters). The cartridge was eluted with 30 mL of 100% hexanes, 2 × 10 mL of 15% ethyl acetate in hexanes (3 × 10 mL for some batches) and 10 mL of 40% ethyl acetate in hexanes. The activity in the first 6 mL of the 40% ethyl acetate in hexanes alone, or in some cases combined with the second 15% fraction, was used for sdAb conjugation. Radiochemical purity of the Boc_2_-*iso*-[^131^I]SGMIB in the different fractions was evaluated using NP-HPLC^26^.

Fractions containing Boc_2_-*iso*-[^131^I]SGMIB were pooled and dried under a gentle flow of nitrogen. Trifluoroacetic acid (TFA, 200 µL) was added, the vial vortexed for 30 s and the reaction mixture was incubated at room temperature for 15 min. Subsequently, TFA was removed under a stream of nitrogen, followed by co-evaporation with ethyl acetate (100 µL × 3). VHH_1028 (222–1150 µg, pH 8.5 borate buffer, 7–9 mg/mL) was added at a 1.2:1 VHH_1028:*iso*-[^131^I]SGMIB molar ratio and the reaction mixture was vortexed for 30 s and incubated at 37 °C for 30 min. The labeled sdAb was isolated by gel filtration over a PD-10 column eluted with PBS and fractions containing the *iso*-[^131^I]SGMIB-VHH_1028 conjugate were combined.

### Quality control

The radiochemical purity of *iso*-[^131^I]SGMIB-VHH_1028 was evaluated by SDS-PAGE/phosphor imaging and its immunoreactive fraction was determined by the Lindmo method^48^ following a reported procedure^49^. *Iso*-[^131^I]SGMIB-VHH_1028 HER2 binding affinity was evaluated using SKOV-3 cells as described^50,51^. Nonspecific binding was measured in parallel assays by co-incubating cells with a 100-fold molar excess of trastuzumab.

### In vitro stability

*Iso*-[^131^I]SGMIB-VHH_1028 (307 MBq) was added to 10 mL of PBS and incubated at 4 °C. Aliquots (1 µL) were analyzed by SDS-PAGE/phosphor imaging at 18, 24, 42 and 48 h.

### Cell culture conditions

SKOV-3 human ovarian carcinoma cells and BT474 human breast carcinoma cells were obtained from the Duke University Cell Culture Facility. SKOV-3 cells were grown in McCoy’s 5A medium containing 10% fetal bovine serum and 1% penicillin–streptomycin. BT474 cells were grown in Dulbecco’s Modified Eagle's Medium (DMEM) containing 10% FBS, supplemented with 10 mg/mL bovine insulin. Cells were cultured at 37 °C in a 5% CO_2_ humidified incubator.

### Cell binding, retention and internalization

The binding and cellular retention of *iso*-[^131^I]SGMIB-VHH_1028 was measured on BT474 cells as described^21,26^. Cells were incubated with *iso*-[^131^I]SGMIB-VHH_1028 at 4 °C for 1 h, unbound activity was removed and uptake of ^131^I was assessed. Then the cells were incubated at 37 °C for 1, 2, 4 and 24 h for measurement of cell-bound and internalized radioactivity. The internalization rate constant (k_e_) for *iso*-[^131^I]SGMIB-VHH_1028 was determined as reported previously^24^. For this, BT474 cells (8 × 10^5^ cells/well/3 mL medium) in 6-well plates were incubated overnight at 37 °C. Medium was removed and replaced with fresh medium containing 1 nmol of *iso*-[^131^I]SGMIB-VHH_1028 and the fraction of unbound, surface-bound and internalized ^211^At activity after incubation at 37 °C for 1, 2, 3, 4, and 5 h was determined as above. In all experiments, nonspecific binding was assessed by co-incubating cells with a 100-fold molar excess of trastuzumab.

### Biodistribution studies

All experimental protocols were reviewed and approved by the Occupational and Environmental Safety Office and the Radiation Safety Committee of Duke University. All methods were carried out in accordance with relevant guidelines and regulations. All experiments involving animals were carried out in accordance with the guidelines and regulations of Institutional Animal Care and Use Committee (IACUC) of Duke University and described in approved protocol Number A246-18-10. All animal studies were performed in accordance with Animal Research: Reporting of In Vivo Experiments (ARRIVE) guidelines. For BT474 xenografts, 4-week old female NOD SCID mice (25 g; Jackson Labs) received subcutaneous implants of estrogen pellets (17β-estradiol, 0.72 mg) in the back of the neck. Two days later, 20 × 10^6^ BT474 cells in 1:1 (v/v) Matrigel® (Corning Inc., NY):tissue culture medium (100 μL) was injected in the shoulder of each mouse. When tumors reached ~ 250 mm^3^ , groups of animals from the same cohort received 100–150 kBq of either *iso*-[^131^I]SGMIB-VHH_1028 or [^131^I]SGMIB-2Rs15d (1 µg sdAb, in 100 µL PBS) via the tail vein. Groups of 5 mice were killed by isoflurane overdose 1, 4, 8, 16 and 24 h for biodistribution analysis. Blood and urine were collected, tumor and other tissues were harvested, and then weighed and counted along with ^131^I injection standards using an automated gamma counter. Results were expressed as percent injected dose (%ID) per organ and per gram of tissue (%ID/g).

Athymic mice were inoculated in the flank with 5 × 10^6^ SKOV-3 cells in 1:1 (v/v) Matrigel®:tissue culture medium (100 μL). When tumors were ~ 80 mm^3^, the biodistribution of *iso*-[^131^I]SGMIB-VHH_1028 and [^131^I]SGMIB-2Rs15d was evaluated individually as described above.

### Radiation dosimetry

The biodistribution data sets were used to calculate radiation dosimetry for *iso*-[^131^I]SGMIB-VHH_1028 and [^131^I]SGMIB-2Rs15d in two potential settings—mouse and human. For the mouse, radiation absorbed doses to tumor and kidney were calculated for a hypothetical 37-MBq (1 mCi) dose in both xenograft models following previously described methods^11,34,52^. Briefly, %ID/g values were corrected for radioactive decay; then linear and nonlinear regression was performed to fit tumor and kidney activity (µCi/g) over time, respectively. Cumulative activity (µCi*h/g) was calculated by trapezoidal integration of these data until negligible radioactivity remained in these tissues. Uniform distribution of ^131^I in tumors was assumed. Radiation absorbed dose (cGy) was calculated by multiplying cumulative activity by the ^131^I equilibrium absorbed dose constant, 0.4313 g*cGy/(µCi*h)^52^. Dose due to ^131^I γ-emissions was not included due to the small volume of these tissues.

Organ and effective doses for both ^131^I-labeled sdAbs were estimated for humans as follows: First, the mouse biodistribution data were used to compute time-integrated activity coefficients (residence times) for selected source organs. The residence times in mouse organs were scaled to correct for the differences in organ masses relative to body weights between species. An estimate of retained total body activity at 1 h post injection showed that a significant fraction of the dosage was excreted by then, suggesting that the mice metabolized both compounds more rapidly than would be anticipated in humans. Therefore, the mouse residence times were adjusted to the equivalent human time scale. The adjusted residence times were used as input to the OLINDA/EXM program (version 1.0). The adult female phantom and a 4.5-h bladder voiding interval were used to compute organ absorbed doses and effective doses (ICRP Report 103 tissue weighting factors).

### Therapeutic efficacy of ***iso***-[^131^I]SGMIB-VHH_1028

The therapeutic efficacy of *iso*-[^131^I]SGMIB-VHH_1028 was evaluated in both single-dose and multiple-dose protocols. *Iso*-[^131^I]SGMIB-VHH_1028 was produced as described and purity of each batch was determined using SDS-PAGE. The single-dose experiment was performed in athymic mice with subcutaneous SKOV-3 xenografts. Mice with ~ 75 mm^3^ tumors were randomized into 4 groups (n = 8), and injected *i.v.* with 10, 28, or 56 MBq, of *iso*-[^131^I]SGMIB-VHH_1028 (42 µg sdAb for all) in 100 µL PBS or PBS alone (100 µL) and designated as the low, medium and high dose, and control group, respectively. Body weight and overall health of the mice were monitored 3 times/week for 87 days. Tumor size was measured 3 times per week using a caliper and the volume calculated according to the formula Volume = Length x Width^2^ × 0.52.

The therapeutic efficacy of multiple doses of *iso*-[^131^I]SGMIB-VHH_1028 was evaluated in NOD SCID mice with subcutaneous BT474 xenografts. Mice with ~ 160 mm^3^ tumors were randomized into 4 groups (n = 12), and injected *i.v*. with 10, 18 or 30 MBq, of *iso*-[^131^I]SGMIB-VHH_1028 in 100 µL PBS (5–23 µg sdAb) or PBS alone on Day 0, 8, 14 and 21. These were designated as the low, medium and high dose, and control groups, respectively. Tumor volume, body weight and overall health were monitored as described above. In both experiments, mice were euthanized if any of the following occurred: (1) tumor volume > 1000 mm^3^, (2) body weight loss > 20%, (3) tumor ulceration or necrosis, or (4) other health conditions that necessitated euthanasia per Duke IACUC policy.

### Statistical analysis

Statistical methods used to analyze the biodistribution and therapeutic efficacy experiments are presented in Supplementary Methods.

## Supplementary Information

Supplementary Information.
