# Supplementary material for: Evaluation of an 131I-labeled HER2-specific single domain antibody fragment for the radiopharmaceutical therapy of HER2-expressing cancers
Source: Sci Rep. 2022 Feb 22;12:3020. doi: 10.1038/s41598-022-07006-9 (PMC8864007; doi:10.1038/s41598-022-07006-9)
Supplement: Supplementary file 1 — Supplementary Information. [file 41598_2022_7006_MOESM1_ESM.pdf]

*Supplemental Information for:*

# Evaluation of an $^{131}$I-labeled HER2-specific single domain antibody fragment for the radiopharmaceutical therapy of HER2-expressing cancers

Yutian Feng[1], Rebecca Meshaw[1], Darryl McDougald[1,2], Zhengyuan Zhou[1], Xiao-Guang Zhao[1], Stephen A. Jannetti[1], Robert E. Reiman[1], Erica Pippen[2], Robin Marjoram[2], Jeffrey L. Schaal[2], Ganesan Vaidyanathan[1] & Michael R. Zalutsky[1]

[1]Department of Radiology, Duke University Medical Center, Durham, NC USA.

[2]Cereius Inc, Durham, NC USA.

## Supplemental Methods

**General.** The reagents used in this work were purchased from Sigma-Aldrich except as noted below. Reagents for cell culture were obtained from Thermo Fisher Scientific (Waltham, MA). Sodium [$^{131}$I]iodide was obtained either from Perkin Elmer (in 0.1 M NaOH, 185 GBq/mg, >18.5 GBq/mL) or from International Isotopes Inc. (in a solution of 0.04 M sodium thiosulfate, 0.2 M NaOH and 0.2 M sodium carbonate, 936 GBq/mg, 236.097 GBq/mL) (Idaho Falls, ID). The synthesis of *iso*-[$^{131}$I]SGMIB and [$^{131}$I]SGMIB, and conjugation of these prosthetic agents to VHH_1028 sdAbs and 2Rs15d, respectively, were performed adapting methods previously [1-3].

The anti-HER2 sdAb 2Rs15d was produced by ATUM (Newark, CA) using published procedures [4, 5] and amino acid sequence [6], and its affinity (2.1 nM), determined by surface plasmon resonance, was consistent with published values [5, 6].

The anti-HER2 mAb, trastuzumab (Roche/Genentech), was obtained from the Duke University Medical Center Pharmacy. High performance liquid chromatography (HPLC) was conducted using two Agilent 1260 Infinity systems equipped with a 1260 Infinity Multiple Wavelength Detector (Santa Clara, CA). For monitoring radioactivity, one system was connected to a Dual Scan-RAM flow activity detector/TLC scanner and the other to a Flow-RAM detector (Lablogic, Tampa, Florida); both the HPLC and the gamma detectors were controlled by LabLogic Laura software. Hydrophobic Interaction Chromatography (HIC) HPLC was utilized for separation of conjugates of sdAbs using a TSKgel Butyl-NPR 2.5 $\mu$m particle-size 4.6 mm ID × 10 cm column (Tosoh Bioscience, Montgomeryville, PA). This column was equilibrated with the following mobile phase:

mobile phase A is 25 mM sodium phosphate buffer and 1.5 M ammonium sulfate (pH=7.0); mobile phase B is 25 mM sodium phosphate buffer and 2-propanol (80% v/v sodium phosphate buffer and 20% v/v 2-propanol) (pH=7.0). The column was eluted with 0 to 100% B in 20 min at a flow rate of 0.5 mL/min. Analysis of the *iso*-SGMIB-VHH_1028 conjugate was done by HPLC using an Agilent PL Multisolvent 20 column eluted with pure Milli-Q® water as the mobile phase. Disposable PD-10 desalting columns for gel filtration were purchased from GE Healthcare (Piscataway, NJ). Radioactivity levels were measured using a CRC-7 dose calibrator (Capintec, Pittsburgh, PA) for higher activities, and either an LKB 1282 (Wallac, Finland) or a Perkin Elmer Wizard II (Shelton, CT) automated gamma counter for lower activities. Mass spectra were obtained using an Advion (Ithaca, NY) ExpressionL CMS LC-MS System attached to an Agilent 1260 infinity HPLC, like the system described above. This mass spectrometer has the capability of determining molecular weights of compounds directly from TLC plates (Plate Express), and by ESI, atmospheric pressure chemical ionization (APCI), and atmospheric solids analysis probe (ASAP).

**VHH_1028.** All experiments described in this section were performed by Cereius, Inc. VHH_1028 was designed to enhance its compatibility for drug development and targeted radionuclide therapy by being engineered to ensure that no lysine residues were present in its CDR loops (Fig. S1). VHH_1028 was purified using Protein A chromatography to high purity (Fig. S1). VHH_1028 has a calculated molecular weight of 12,844 Da and an isoelectric point of 9.01.

VHH_1028 was recombinantly encoded in the pD2610-v5 plasmid vector (ATUM) with the signal peptide azurocidin and cloned into the NEB® 5α competent *E. coli* (New England Biosciences). *E. coli* were inoculated in 2xYT media (ThermoFisher Scientific) with 45 $\mu$g/mL kanamycin and grown overnight. The *E.coli* plasmids were then harvested using the EndoFree Plasmid Maxi Kit and protocol (Qiagen). The plasmids were complexed to ExpiFectamine® reagent (ThermoFisher) and then transiently transfected into ExpiCHO-S mammalian cells (ThermoFisher Scientific) at a concentration of 1 $\mu$g/mL per tissue culture volume. ExpiCHO-S cells were then maintained under standard conditions of 37°C and 8% $CO_2$ for 10 days before the protein was harvested. For harvesting, cells were isolated out of culture by centrifugation at 4000 rcf for 15 min. The resulting supernatant containing secreted $V_HH$ was then purified using 1 mL Pierce™ Protein A chromatography cartridges (Thermo Fisher Scientific).

Purity of the protein was assessed using SDS-PAGE gels (BioRad) under reducing and non-reducing conditions. Purity was likewise assessed via size exclusion HPLC at 220 nm on an AdvanceBio SEC 120A column (Agilent Technologies). Before undertaking further experiments, protein purity was determined to be greater than 95% by both methods. Endotoxin levels were next determined utilizing the Endosafe® Nexgen PTX (Charles River) to ensure endotoxin levels were lower than $<0.50$ EU/mg. Last, the mass of the protein was verified using ESI-LCMS under reducing conditions with PNGase F on a 6530 Accurate-Mass A-TOF MC/MS (Agilent Technologies at ATUM). The resulting protein was then formulated in PBS at a concentration of 2 mg/mL and stored at -80°C.

The affinity, specificity, and cross-reactivity of VHH_1028 was first examined in a series of colorimetric, indirect ELISA assays. Briefly, ELISA plates for each antigen were created by preparing each antigen at a concentration of 5 $\mu$g/mL, adding 50 $\mu$L to each well in a 96-well Immulon 4 HBX plate (Thermo Fisher Scientific), covering the plates, and then incubating at room temperature for four hours. After four hours, the plates were washed with PBS and then 200 $\mu$L per well of 1% BSA (Sigma Aldrich) blocking solution was added. The plates were then blocked overnight at 4°C. Human antigens consisted of recombinant human HER2-Fc (#10004-H02H, Sino Biologic), human HER2-$His_6$ (#10126-ER-050, R&D Systems), the Fc fragment of human IgG (#009-000-008, Jackson Immunology Research Lab), recombinant human EGFR-Fc (#10001-H02H, Sino Biologic), recombinant human HER3-Fc (#10201-H02H, Sino Biologic), and recombinant HER4-Fc (#10363-H02H, Sino Biologic). Cross-species HER2 recombinant antigen were likewise sourced for mouse (#50714-M02H, Sino Biologic), rat (#80079-R02H, Sino Biologic), canine (#70024-D08H, Sino Biologic), cynomolgus (#90295-C02H, Sino Biologic), and rhesus HER2 (#90020-K02H, Sino Biologic).

To evaluate binding affinity, VHH_1028 was plated in serial log dilutions, in triplicates of 50 $\mu$L/well, ranging from 0.0001 nM to 10,000 nM. Plates were incubated for 60 min on an orbital shaker at room temperature to allow binding. Plates were then washed three times with PBS before adding 50 $\mu$L/well of MonoRab™ Rabbit Anti-Camelid VHH polyclonal antibody cocktail with HRP (#A02016-200, GenScript) prepared at a 1:3000 dilution in PBS/Tween. Plates were incubated for 30 min and then washed with PBS. Ultra TMB (#34029, Thermo Fisher Scientific) was next added, incubated for 3 min, and then

quenched with 2 M $H_2SO_4$. Colorimetric absorbance of each plate well was measured at 450 nm using a Varioskan Lux microplate reader (Thermo Fisher Scientific). The binding constant was evaluated using the nonlinear regression analysis package of GraphPad Prism 9.1, as calculated with a four-parameter, variable dose-response equation. In addition to the technical replicates within each assay, the ELISA assays were replicated 3-4 times for each antigen.

The binding affinity of VHH_1028 for the human HER2 receptor was assessed using two different recombinant antigen display modalities sourced from two different vendors. In both instances, a sub-nanomolar affinity for human HER2 was evidenced regardless of vendor or recombinant antigen display method. Specifically, $EC_{50}$ against the HER2-Fc fusion was found to be 0.77 ± 0.19 nM, while the $EC_{50}$ was 0.69 ± 0.18 nM for the R&D Systems antigen comprising only the extracellular domain of the HER2 receptor (Fig. S2). VHH_1028 did not exhibit any non-specific affinity for any of the other receptors in the human ErbB family other than the low background associated with IgG Fc fragment.

Next, the cross-reactivity of VHH_1028 was evaluated against 5 animal species variants of HER2 (Fig. S2). VHH_1028 showed no affinity for either mouse or rat HER2. However, it demonstrated strong affinity for HER2 receptors in canine and two species of non-human primate. The $EC_{50}$ values were 1.09 nM, 1.19 nM, and 1.18 nM for canine, cynomolgus, and rhesus species, respectively. A full breakdown of all affinity values for all molecular targets also can be found in Fig S2.

**Statistical Analysis.** Data are presented as mean ± standard deviation. Statistical significance for the biodistribution experiments was evaluated using a paired two-tailed

Student *t*-test in GraphPad Prism 8. Differences with a $P$ value $< 0.05$ were considered to be statistically significant.

Statistical and data analysis for the therapeutic efficacy studies was performed as follows: for each group, the average body weight and the standard deviation were calculated and plotted versus time. Normalized average body weight was calculated by setting the initial average body weight on the day of treatment (Day 0) to 100%. Animals were excluded at the day of death. Two-tailed paired *t* tests were conducted for the normalized average body weight between each group, and tests were only conducted for days when alive animals were present in each group. Average tumor volumes and standard deviations were calculated for each group and plotted versus time. When animals died or were euthanized, their tumor volume at the day of death was carried forward after the day of death for the calculation of average tumor volume. Two-tailed paired *t* tests were conducted for the average tumor volumes between each group, and tests were only conducted for days when animals remained alive in each group. Differences with a $P$ value $< 0.05$ were considered statistically significant. For survival data, log-rank (Mantel-Cox) test, log-rank test for trend, and Gehan-Breslow-Wilcoxon test were conducted. Maximum tumor response waterfall plot was generated for the multiple-dose study by recording the maximum tumor response (growth or inhibition) for each mouse during the experimental period.

**Supplementary Table S1**. Biodistribution of *iso*-[$^{131}$I]SGMIB-VHH_1028 in athymic mice bearing SKOV-3 xenografts.

| | Percent injected dose per gram of tissue[a] | | | | | |
|---|---|---|---|---|---|---|
| Tissues | 1 h | 2 h | 4 h | 8 h | 16 h | 24 h |
| Liver | 1.0 ± 0.1 | 0.8 ± 0.1 | 0.2 ± 0.0 | 0.1 ± 0.0 | 0.0 ± 0.0 | 0.0 ± 0.0 |
| Spleen | 0.4 ± 0.2 | 0.5 ± 0.1 | 0.1 ± 0.0 | 0.1 ± 0.0 | 0.0 ± 0.0 | 0.1 ± 1.0 |
| Lungs | 1.2 ± 0.1 | 0.9 ± 0.1 | 0.3 ± 0.1 | 0.2 ± 0.1 | 0.1 ± 0.0 | 0.1 ± 0.0 |
| Heart | 1.5 ± 2.3 | 0.3 ± 0.0 | 0.1 ± 0.0 | 0.0 ± 0.0 | 0.1 ± 0.0 | 0.0 ± 0.0 |
| Kidneys | 35.6 ± 11.0 | 10.0 ± 2.7 | 2.4 ± 0.7 | 0.5 ± 0.1 | 0.2 ± 0.0 | 0.1 ± 0.0 |
| Stomach | 1.0 ± 0.2 | 1.0 ± 0.2 | 0.2 ± 0.1 | 0.2 ± 0.1 | 0.0 ± 0.0 | 0.0 ± 0.0 |
| Thyroid[b] | 0.1 ± 0.0 | 0.2 ± 0.0 | 0.1 ± 0.0 | 0.1 ± 0.0 | 0.2 ± 0.1 | 0.2 ± 0.1 |
| Sm. Int. | 0.7 ± 0.2 | 0.8 ± 0.2 | 0.2 ± 0.0 | 0.1 ± 0.0 | 0.0 ± 0.0 | 0.0 ± 0.0 |
| Lg. Int. | 0.3 ± 0.1 | 0.3 ± 0.1 | 1.0 ± 0.3 | 0.5 ± 0.3 | 0.1 ± 0.0 | 0.1 ± 0.0 |
| Muscle | 0.3 ± 0.1 | 0.1 ± 0.0 | 0.0 ± 0.0 | 0.0 ± 0.0 | 0.0 ± 0.0 | 0.0 ± 0.0 |
| Blood | 0.7 ± 0.2 | 0.4 ± 0.0 | 0.1 ± 0.0 | 0.1 ± 0.0 | 0.1 ± 0.0 | 0.0 ± 0.0 |
| Bone | 0.6 ± 0.2 | 0.2 ± 0.0 | 0.1 ± 0.0 | 0.0 ± 0.0 | 0.0 ± 0.0 | 0.0 ± 0.0 |
| Brain | 0.0 ± 0.0 | 0.0 ± 0.0 | 0.0 ± 0.0 | 0.0 ± 0.0 | 0.0 ± 0.0 | 0.0 ± 0.0 |
| Tumor | 5.6 ± 1.9 | 4.8 ± 1.0 | 7.0 ± 2.6 | 4.7 ± 2.5 | 6.5 ± 1.2 | 3.1 ± 1.3 |

[a]Mean ± SD (n=5); [b]% ID/organ

**Supplementary Table S2**. Biodistribution of [$^{131}$I]SGMIB-2Rs15d in athymic mice bearing SKOV-3 xenografts.

| | Percent injected dose per gram of tissue[a] | | | | | |
|---|---|---|---|---|---|---|
| Tissues | 1 h | 2 h | 4 h | 8 h | 16 h | 24 h |
| Liver | 0.5 ± 0.1 | 0.3 ± 0.1 | 0.2 ± 0.0 | 0.1 ± 0.0 | 0.0 ± 0.0 | 0.0 ± 0.0 |
| Spleen | 0.2 ± 0.0 | 0.1 ± 0.0 | 0.1 ± 0.0 | 0.1 ± 0.0 | 0.0 ± 0.0 | 0.0 ± 0.0 |
| Lungs | 0.4 ± 0.1 | 0.3 ± 0.1 | 0.2 ± 0.1 | 0.1 ± 0.0 | 0.0 ± 0.0 | 0.1 ± 0.0 |
| Heart | 0.2 ± 0.0 | 0.1 ± 0.0 | 0.1 ± 0.0 | 0.0 ± 0.0 | 0.0 ± 0.0 | 0.0 ± 0.0 |
| Kidneys | 49.4 ± 14.1 | 22.6 ± 3.8 | 4.7 ± 1.5 | 1.6 ± 0.4 | 0.3 ± 0.0 | 0.3 ± 0.1 |
| Stomach | 0.4 ± 0.1 | 0.5 ± 0.3 | 0.1 ± 0.0 | 0.2 ± 0.1 | 0.0 ± 0.0 | 0.0 ± 0.0 |
| Thyroid[b] | 0.1 ± 0.1 | 0.1 ± 0.0 | 0.0 ± 0.0 | 0.1 ± 0.0 | 0.1 ± 0.0 | 0.1 ± 0.1 |
| Sm. Int. | 0.5 ± 0.1 | 0.3 ± 0.2 | 0.1 ± 0.0 | 0.1 ± 0.0 | 0.0 ± 0.0 | 0.0 ± 0.0 |
| Lg. Int. | 0.1 ± 0.0 | 0.4 ± 0.2 | 0.5 ± 0.2 | 0.3 ± 0.2 | 0.0 ± 0.0 | 0.0 ± 0.0 |
| Muscle | 0.1 ± 0.0 | 0.1 ± 0.0 | 0.0 ± 0.0 | 0.0 ± 0.0 | 0.0 ± 0.0 | 0.0 ± 0.0 |
| Blood | 0.3 ± 0.0 | 0.1 ± 0.0 | 0.1 ± 0.0 | 0.1 ± 0.0 | 0.0 ± 0.0 | 0.0 ± 0.0 |
| Bone | 0.2 ± 0.0 | 0.1 ± 0.0 | 0.0 ± 0.0 | 0.0 ± 0.0 | 0.0 ± 0.0 | 0.0 ± 0.0 |
| Brain | 0.0 ± 0.0 | 0.0 ± 0.0 | 0.0 ± 0.0 | 0.0 ± 0.0 | 0.0 ± 0.0 | 0.0 ± 0.0 |
| Tumor | 3.5 ± 1.6 | 4.6 ± 1.6 | 3.1 ± 1.6 | 3.4 ± 1.3 | 3.1 ± 0.8 | 3.3 ± 2.6 |

[a]Mean ± SD (n=5); [b]% ID/organ

**Supplementary Table S3**. Radiation dose estimates of [$^{131}$**I**]SGMIB-2Rs15d and *iso*-[$^{131}$**I**]SGMIB-VHH_1028 to different organs for adult female human based on OLINDA calculations. Calculations were based on biodistribution data in Table 1 and 2.

| Target Organ | [$^{131}$I]SGMIB-2Rs15d Dose (mSv/MBq) | *iso*-[$^{131}$I]SGMIB-VHH_1028 Dose (mSv/MBq) |
|---|---|---|
| Adrenals | 6.35E-02 | 8.43E-02 |
| Brain | 4.22E-02 | 6.62E-02 |
| Breasts | 4.18E-02 | 6.36E-02 |
| Gallbladder Wall | 6.25E-02 | 8.54E-02 |
| LLI Wall | 6.53E-02 | 2.36E-01 |
| Small Intestine | 8.05E-02 | 1.36E-01 |
| Stomach Wall | 5.42E-02 | 8.11E-02 |
| ULI Wall | 5.98E-02 | 1.80E-01 |
| Heart Wall | 5.44E-02 | 7.98E-02 |
| Kidneys | 4.42E-01 | 2.50E-01 |
| Liver | 1.24E-01 | 8.22E-02 |
| Lungs | 1.00E-01 | 1.13E-01 |
| Muscle | 4.93E-02 | 7.43E-02 |
| Ovaries | 6.55E-02 | 1.03E-01 |
| Pancreas | 6.13E-02 | 8.57E-02 |
| Red Marrow | 4.35E-02 | 6.52E-02 |
| Osteogenic Cells | 9.92E-02 | 1.53E-01 |
| Skin | 3.96E-02 | 6.08E-02 |
| Spleen | 5.73E-02 | 8.08E-02 |
| Thymus | 4.88E-02 | 7.47E-02 |
| Thyroid | 4.42E-02 | 6.88E-02 |
| Urinary Bladder Wall | 8.60E-01 | 9.97E-01 |
| Uterus | 7.85E-02 | 1.14E-01 |
| Total Body | 5.54E-02 | 7.93E-02 |
| Effective Dose | 0.105 | 0.148 |

**Supplementary Table S4**. Experimental details for the single-dose therapy study using athymic mice with SKOV-3 xenografts for *iso*-[$^{131}$I]SGMIB-VHH_1028.

| Group | Initial Tumor Volume (mm$^3$) | $^{131}$I Dose (MBq) | VHH Dose (µg) |
|---|---|---|---|
| Control (PBS) | 78.1 ± 47.6 | 0 | 0 |
| Low Dose | 84.9 ± 35.1 | 10.0 ± 0.7 | 42 |
| Medium Dose | 63.1 ± 29.4 | 28.2 ± 2.3 | 42 |
| High Dose | 74.9 ± 26.8 | 55.7 ± 11.7 | 42 |

**Supplementary Table S5**. Experiment details for the multi-dose therapy study using SCID mice with BT474 xenografts for *iso*-[$^{131}$I]SGMIB-VHH_1028.

| Group | Initial Tumor Volume ($mm^3$) | Day 0: 1st treatment | | Day 8: 2nd treatment | | Day 14: 3rd treatment | | Day 21: 4th treatment | |
|---|---|---|---|---|---|---|---|---|---|
| | | $^{131}$I Dose (MBq) | VHH Dose (μg) | $^{131}$I Dose (MBq) | VHH Dose (μg) | $^{131}$I Dose (MBq) | VHH Dose (μg) | $^{131}$I Dose (MBq) | VHH Dose (μg) |
| Control (PBS) | 155.9 ± 57.9 | 0 | 0 | 0 | 0 | 0 | 0 | 0 | 0 |
| High Dose | 167.8 ± 65.2 | 30.2 | 18.6 | 31.2 | 14.4 | 30 | 13.6 | 31.7 | 22.9 |
| Medium Dose | 174.0 ± 66.6 | 17.9 | 10.9 | 16.5 | 7.6 | 18.4 | 7.9 | 16.3 | 11.8 |
| Low Dose | 162.5 ± 58.4 | 10 | 6.4 | 10.3 | 4.8 | 10.4 | 4.6 | 10.3 | 7.5 |

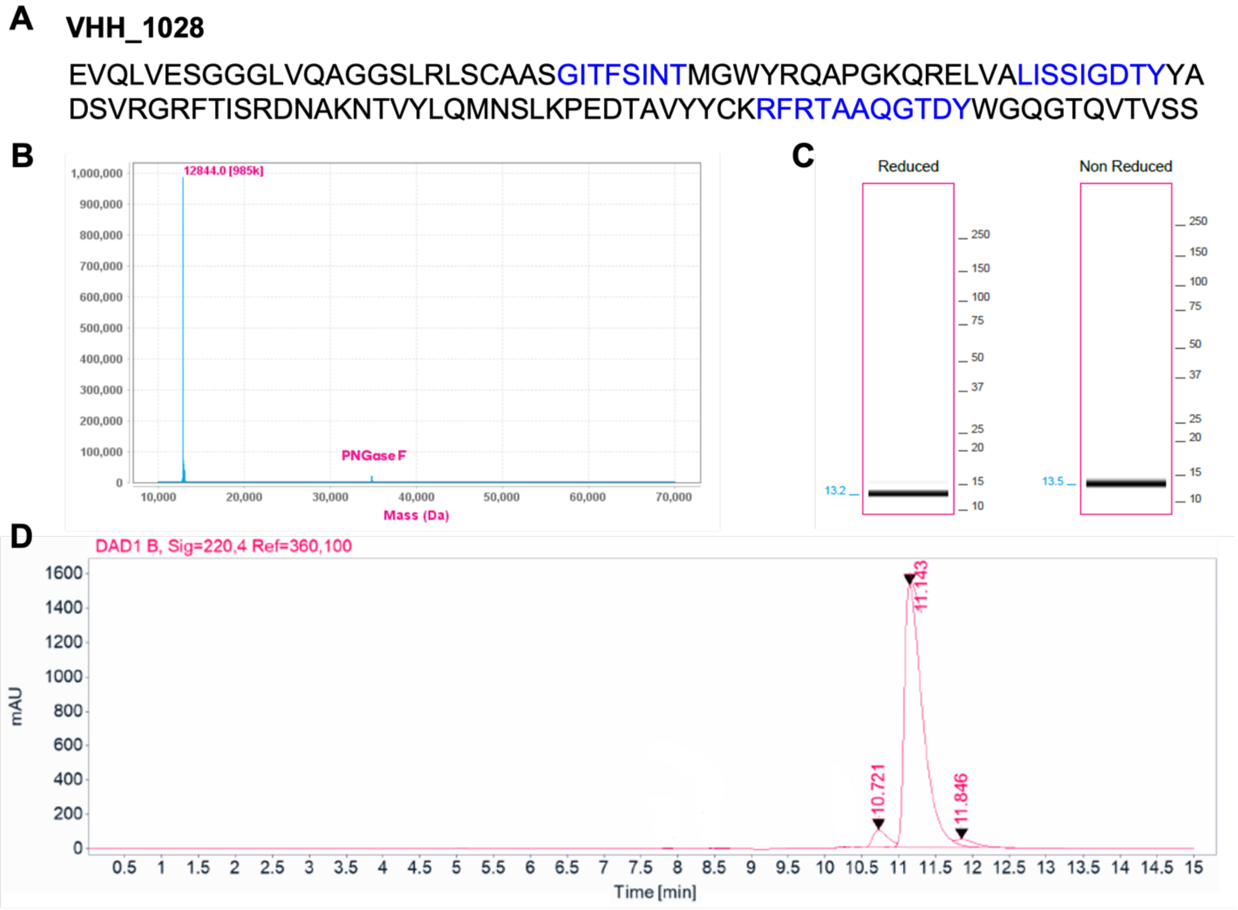


**Supplementary Figure S1**. Protein expression and physical characterization of VHH_1028. (A) Linear amino acid sequence with CDR regions indicated in blue font. (B) ESI-LCMS after PNGase F treatment, verifying a molecular weight of 12844.0 Da. (C) SDS PAGE gel analysis of protein purity under reducing and non-reducing conditions. (D) HPLC trace of VHH after expression in ExpiCHO-s cells and purification with Protein A.

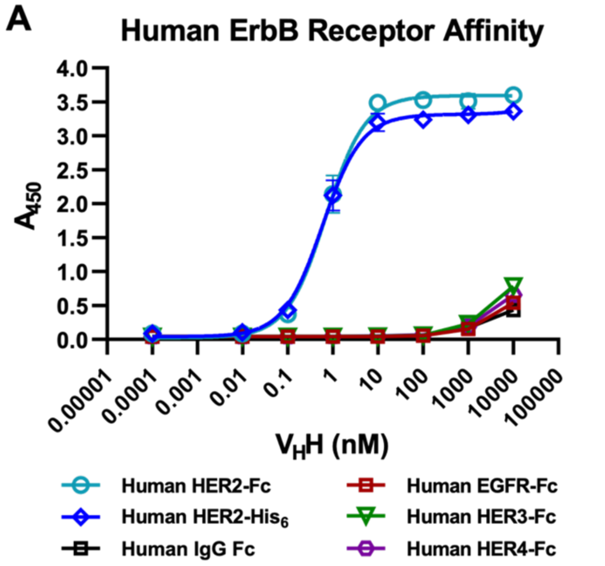


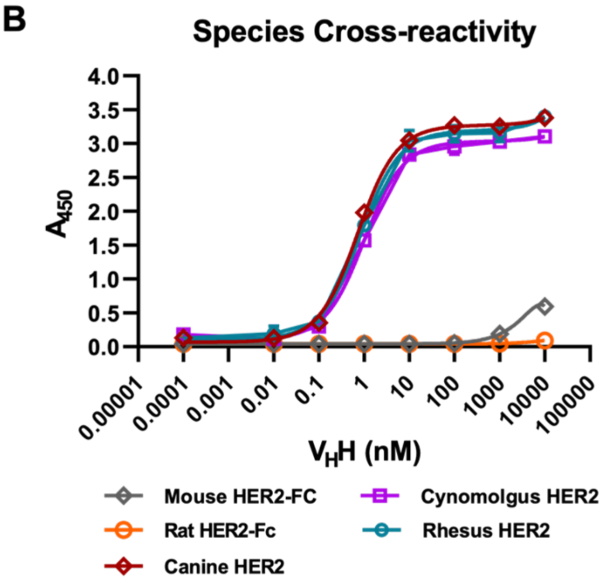


**C** **VHH_1028 Affinity Values**

| ErbB Receptor | $EC_{50}$ (nM) |
|---|---|
| Human HER2-Fc | 0.77 ±0.19 |
| Human HER2-$His_6$ | 0.69 ±0.18 |
| Human EGFR-Fc | > 10,000 |
| Human HER3-Fc | > 10,000 |
| Human HER4-Fc | > 10,000 |
| Mouse HER2-Fc | > 10,000 |
| Rat HER2-Fc | > 10,000 |
| Canine HER2-Fc | 1.09 ±0.40 |
| Cynomolgus HER2-Fc | 1.19 ±0.29 |
| Rhesus HER2-Fc | 1.18 ±0.31 |
| Human IgG Fc | > 10,000 |

**Supplementary Figure S2:** ELISA evaluation of VHH_1028 affinity and specificity to ErbB receptors. (A) Affinity was assessed across all major human ErbB family receptors, demonstrating strong affinity and specificity for HER2. (B) Cross-reactivity of VHH_1028 with HER2 expressed in other species. Cross-reactivity was not found with rodent HER2, but high affinity was maintained for non-human primate and canine antigens. (C) Measured VHH_1028 $EC_{50}$ values.

## VHH_1028

- Immobilization: 651 RU
- On-rate ($k_a$) = 3.468E+06 $M^{-1}s^{-1}$
- Off-rate ($k_d$) = 7.178E-04 $s^{-1}$
- **Affinity ($K_d$) = 2.070E-10 M**
- **Affinity ($K_d$) = 0.21 nM**
- Fit to 1:1 Binding Model
- $Chi^2$ = 0.0643

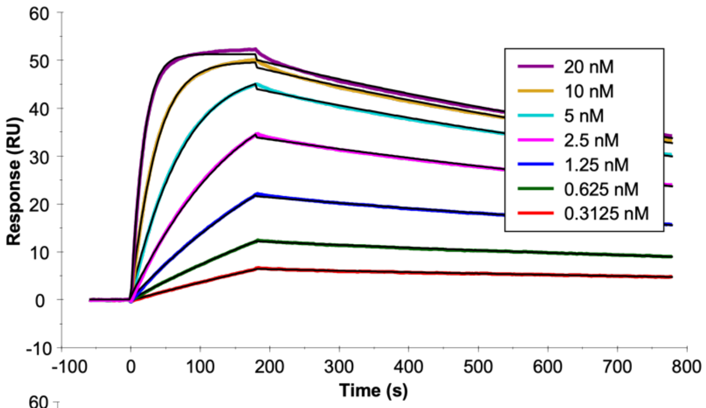


## *iso*-SGMIB-VHH_1028

- Immobilization: 651 RU
- On-rate ($k_a$) = 3.440E+06 $M^{-1}s^{-1}$
- Off-rate ($k_d$) = 7.104E-04 $s^{-1}$
- **Affinity ($K_d$) = 2.065E-10 M**
- **Affinity ($K_d$) = 0.21 nM**
- Fit to 1:1 Binding Model
- $Chi^2$ = 0.0639

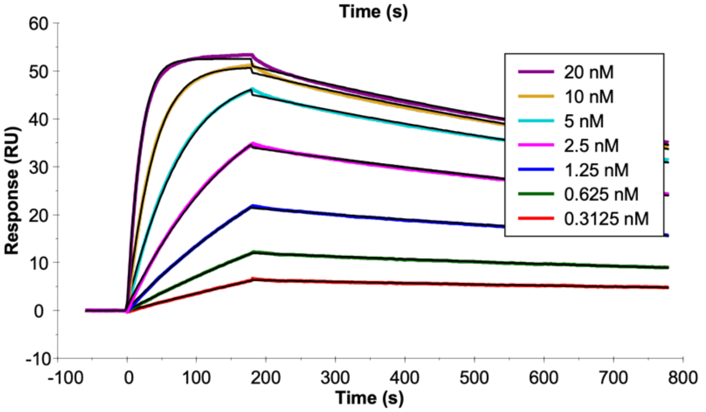


**Supplementary Figure S3**. Surface plasmon resonance sensograms showing dose response curves and kinetic profiles for the binding of varying concentrations of (A) VHH_1028 and (B) the non-radioactive *iso*-SGMIB-VHH_1028 conjugate to HER2-Fc.

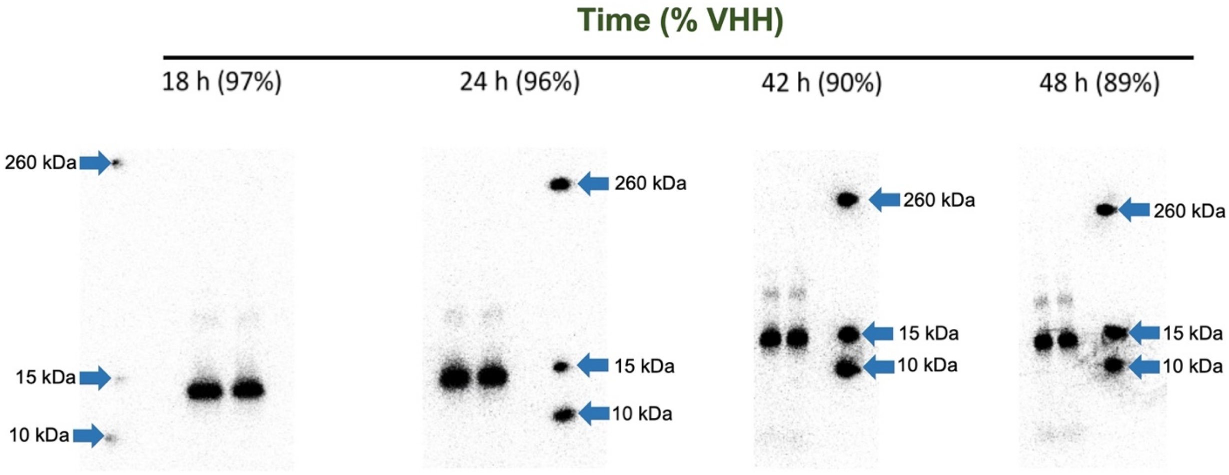


**Supplementary Figure S4.** *In vitro* stability of *iso*-[$^{131}$I]SGMIB-VHH_1028 in PBS. For this study, 370 MBq of *iso*-[$^{131}$I]SGMIB-VHH_1028 was incubated in 10 mL of PBS and stability determined using SDS-PAGE after 18, 24, 42 and 48 h incubation. Molecular weight standards were run at each time point and positions of 10, 15 and 260 kDa bands indicated by blue arrows.

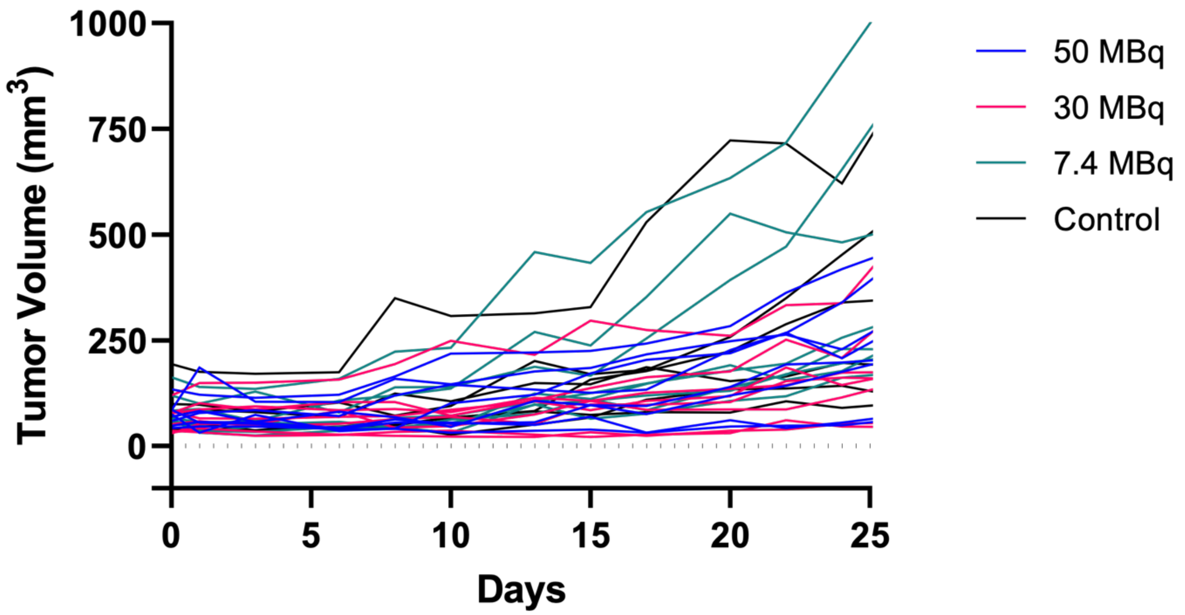


**Supplementary Figure S5.** Tumor volumes in individual athymic mice with subcutaneous SKOV3 xenografts as a function of time after treatment with *iso*-[$^{131}$I]SGMIB-VHH_1028 or PBS control.

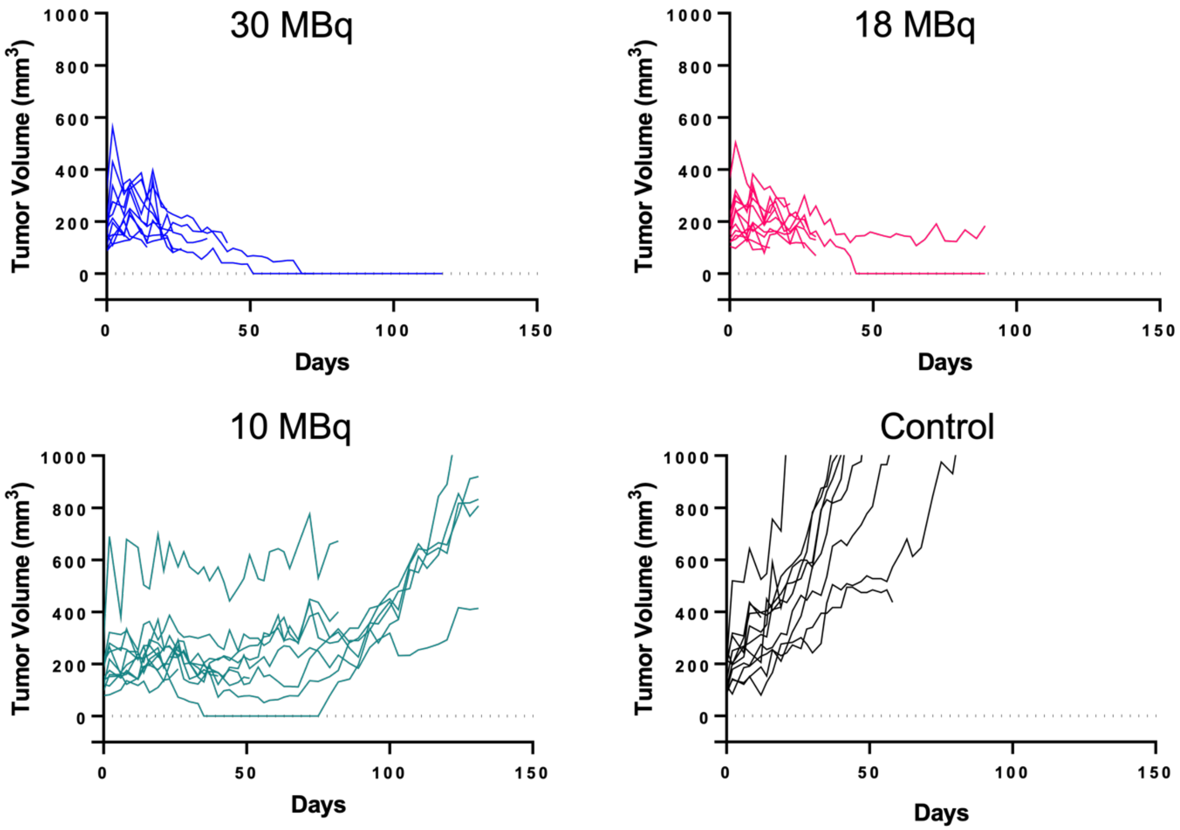


**Supplementary Figure S6.** Tumor volumes in individual SCID mice with subcutaneous BT474 xenografts as a function of time after four treatments with *iso*-[$^{131}$I]SGMIB-VHH_1028 or PBS control.

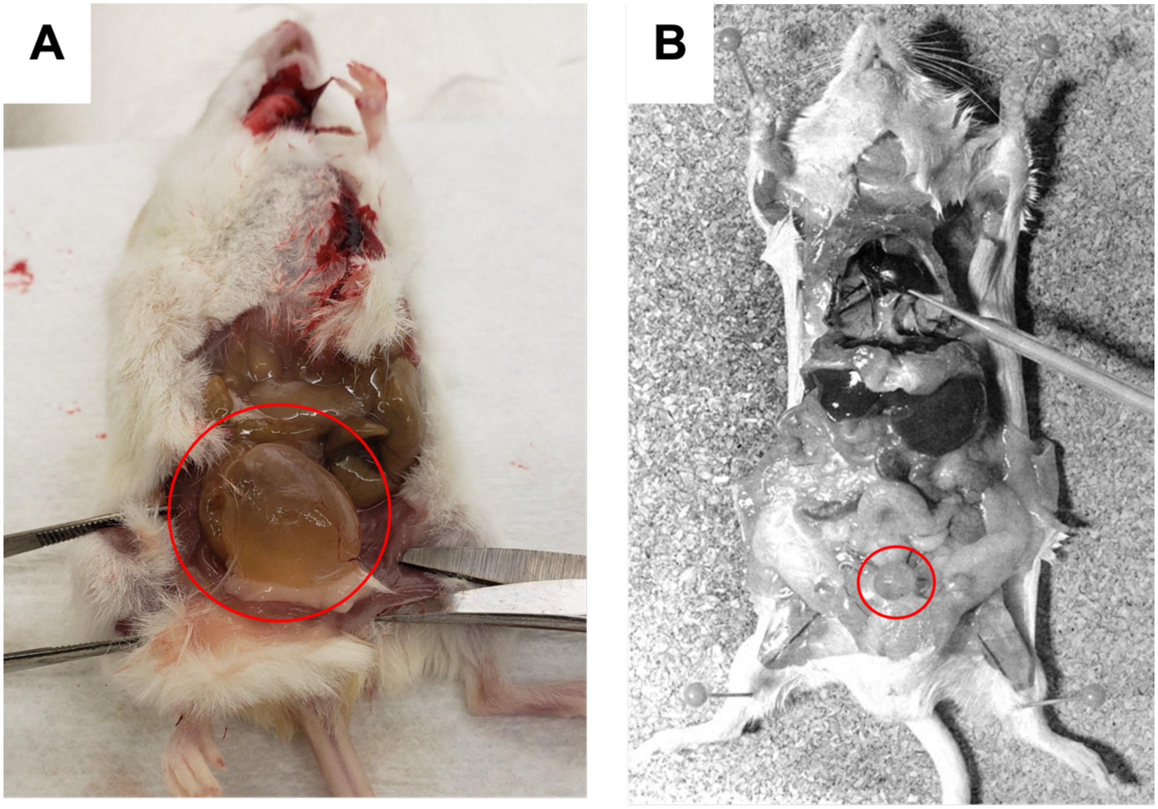


**Supplementary Figure S7**. (A) A mouse implanted with a 0.72 mg estrogen pellet that was subsequently treated with PBS yet developed urolithiasis and died due to a blocked bladder. (B) A comparative bladder found in a healthy mouse that did not receive an estrogen pellet implant. Bladders are circled in red.

## Supplemental References


1. Vaidyanathan, G. & M.R. Zalutsky, Synthesis of N-succinimidyl 4-guanidinomethyl-3-[*I] iodobenzoate: a radio-iodination agent for labeling internalizing proteins and peptides. *Nature protocols*, 2007. **2**(2): p. 282-286.
2. Choi, J., *et al.*, Astatine-211 labeled anti-HER2 5F7 single domain antibody fragment conjugates: Radiolabeling and preliminary evaluation. *Nucl. Med. Biol.*, 2018. **56**: p. 10-20.
3. Feng, Y., *et al.*, Site-specific radioiodination of an anti-HER2 single domain antibody fragment with a residualizing prosthetic agent. *Nucl. Med. Biol.*, 2021. **92**: p. 171-183.
4. Xavier, C., *et al.*, Synthesis, preclinical validation, dosimetry, and toxicity of 68Ga-NOTA-anti-HER2 Nanobodies for iPET imaging of HER2 receptor expression in cancer. *J. Nucl. Med.*, 2013. **54**(5): p. 776-784.
5. Vaneycken, I., *et al.*, Preclinical screening of anti-HER2 nanobodies for molecular imaging of breast cancer. *The FASEB J.*, 2011. **25**(7): p. 2433-2446.
6. Devoogdt, N., De Vos, J., D'Huyvetter, M. & Lahoutte, T. Radiolabelled antibody fragments for use in the prevention and/or treatment of cancer. *United States Patent Number* US 9,855,348 B2 (2018).
